# Co‐Registered Eye‐Movements and Brain Potentials Reveal Multiple Effects of Context Across the Visual Field in Natural Reading

**DOI:** 10.1111/psyp.70173

**Published:** 2025-11-17

**Authors:** Allyson Copeland, Brennan R. Payne

**Affiliations:** ^1^ Department of Psychology University of Utah Salt Lake City Utah USA; ^2^ Department of Communication Sciences and Disorders University of Utah Salt Lake City Utah USA; ^3^ Interdepartmental Neuroscience Program University of Utah Salt Lake City Utah USA

**Keywords:** anterior positivity, FRPs, LPC, N400, natural reading, parafoveal processing, semantic processing

## Abstract

This study investigates how expectancy and plausibility influence behavioral and neural measures of language processing during naturalistic reading comprehension. Prior event‐related potential (ERP) studies show evidence of distinct post‐N400 positivities to violations of semantic expectancy and plausibility using artificial serial presentation but have yet to establish these phenomena during naturalistic reading. Therefore, we recorded simultaneous eye movements and EEG while participants read highly constraining sentences with expected, unexpected (but plausible), and anomalous target words. Time locked to the pre‐target word, we observed a contextually graded parafoveal N400 effect. The N400 was facilitated (i.e., reduced) when the word was subsequently fixated, suggesting trans‐saccadic integration of semantic features. At target fixation, we also observed a late anteriorly distributed positivity to unexpected target words and a posteriorly distributed positivity to anomalous target words, effects that were not clearly present when time locked to the pre‐target word. Eye‐tracking (ET) measures show that readers were sensitive to both expectancy and plausibility at target fixation. In conclusion, we show that readers can begin accessing semantic information in parafoveal vision, but higher‐level semantic processing may require the orchestration of both parafoveal and foveal representations.

## IMPACT STATEMENT

With a natural reading co‐registration paradigm, we demonstrate the first contextually graded parafoveal N400 effect and foveal anterior positivity to unexpected targets within a highly constraining sentence context. These findings are consistent with prior ERP work (but contradict some ET literature) showing that readers engage in some forms of predictive processing before direct fixation at levels beyond mere orthographic match, but that other predictive processes (e.g., processing prediction violations) may require direct fixation.

## 1 Introduction

Extracting coherent messages from text during reading hinges on our ability to quickly integrate incoming sensory information across the visual field with prior information, our existing world knowledge, and current goals. Much psycholinguistic research has focused on understanding the influence of prior context on lexical and semantic word feature activation and integration in reading, using tools such as electroencephalography (EEG) and eye‐tracking (ET). These methods have yielded several divergent findings with respect to both the time course of word processing and the language system's sensitivity to semantic processing violations (cf. Federmeier 2022; Frisson et al. 2017).

Recent technological developments allow for the co‐registration of ET and EEG to estimate *fixation‐related brain potentials* (FRPs; Dimigen et al. 2011; Schotter et al. 2025), offering strong promise towards understanding the neural bases of context processing in naturalistic reading and the unprecedented opportunity to bridge theoretical gaps between these literatures. While the growing co‐registration literature has replicated evidence of N400 modulations in natural reading (e.g., Dimigen et al. 2011; Milligan et al. 2023), very little work has systematically explored activity following the N400. In the ERP literature, distinct anterior and posterior post‐N400 positivities have been elicited to violations of predictability and plausibility, respectively (Delong et al. 2011, 2014; Van Petten and Luka 2012), but evidence for an anterior positivity to prediction violations in natural reading is missing from the literature. This is important since eye‐tracking studies typically fail to show evidence for behavioral sensitivity to plausible prediction violations (Frisson et al. 2017; Luke and Christianson 2016). Thus, a major goal of the current study was to use co‐registration methods to examine simultaneous behavioral and electrophysiological responses to context processing across the visual field in naturalistic reading.

### 1.1 Predictability and Plausibility Effects on ERPs

Some of the strongest evidence for context processing in word reading is from studies measuring ERP components elicited during single‐word reading via rapid serial visual presentation (RSVP; for reviews see Federmeier 2022; Van Petten and Luka 2012). From this literature, we focus on the N400 and two post‐N400 positivities. We refer to these late positivities as the *anterior positivity* (also referred to as the anterior post‐N400 positivity, late anterior positivity, frontal positivity, and late frontal positivity across the literature) and the *late positive component* or LPC (also referred to as the late positive complex, late posterior positivity, semantic P600, posterior post‐N400 positivity).

Remarkably, readers are sensitive to contextual information even before they have made a direct fixation onto a word. A number of studies have adopted a visual hemi‐field flanker RSVP paradigm, developed by Barber et al. (2011), where words are presented in serial triads, with the prior and upcoming words presented in left and right parafoveal vision, respectively. Flanker RSVP allows readers to extract upcoming word information through the parafovea, while still maintaining the beneficial experimental control of RSVP paradigms. Although this paradigm is still quite unnatural (removing the ability to move the eyes, fixate for variable amounts of time, skip words, or re‐read), flanker RSVP has allowed for direct insights into the neural correlates of word processing across the visual field during reading. For example, Stites et al. (2017) show evidence for graded semantic parafoveal processing on the N400 (see also C. Li et al. 2023; N. Li et al. 2024; Kornrumpf et al. 2016; Payne and Federmeier 2017; Payne et al. 2019). The N400 is a centro‐posteriorally distributed negativity peaking around 400 ms after stimulus onset (in standard RSVP) elicited by any meaningful (or potentially meaningful) stimulus and functionally reflects a domain‐general, multi‐modal index of the ease of semantic access (Federmeier 2022). Critically, the N400 strongly relates to the probability of encountering semantic features of likely upcoming words based on prior context. Stites et al. (2017) observed a contextually graded *parafoveal* N400 effect: highly constraining sentences with expected targets facilitated the parafoveal N400 compared to low‐constraint sentences with the same targets. Moreover, unexpected words in highly constraining contexts showed a larger N400 and anomalous words showed the largest (most negative) N400. This graded N400 effect suggests the parafoveal N400 was not driven by simple mismatch between expectations and initial orthographic features of the parafoveal word. This expectancy effect was attenuated when readers subsequently made a direct target word fixation. This reduced target N400 after successful parafoveal semantic processing has been replicated by Payne et al. (2019) using flanker RSVP and Milligan et al. (2023) with a modified flanker RSVP task. Collectively, these parafoveal N400 and foveal attenuation effects suggest that readers initiate access of semantic information when words are visible parafoveally, and these processes continue at foveation. Whether these facilitative effects extend to naturalistic reading remains a critical and important question.

In addition to the relatively rapid time‐course of semantic access observed for the N400, subsequent post‐N400 late positivities have been studied to understand the influence of violations of semantic predictability and plausibility on brain activity during sentence comprehension. In general, theories about late positivities indicate they reflect later‐stage and higher‐order processes than the N400. DeLong et al. (2014) first evaluated with RSVP these post‐N400 positivities simultaneously for anterior and posterior regions using constraining sentences with expected, unexpected but plausible, and semantically anomalous targets. Specifically, they showed a late anteriorly distributed positivity to plausible *prediction* violations (e.g., “She shut the front window and locked the back *room*…”) and a distinct posteriorly distributed positivity to *plausibility* violations (“…locked the back *note*…”; DeLong et al. 2014). This general pattern has been replicated and extended in a number of subsequent studies (C. Li et al. 2023; Kuperberg et al. 2020; Quante et al. 2018; Brothers et al. 2020), suggesting that these two late positivities may functionally reflect dissociable and (at least partially) independent processes.

Some frameworks posit that the anterior positivity reflects *processing consequences* or costs associated with encountering a plausible word that disconfirms previously pre‐activated or predicted information. For example, this effect has been argued to reflect the need to suppress a predicted but never observed lexical candidate (e.g., Ness and Meltzer‐Asscher 2018; Ryskin and Nieuwland 2023). This is consistent with a broader developing literature suggesting that domain‐general executive and cognitive control mechanisms must be engaged for lexical inhibition after encountering semantic violations (Sánchez‐Meléndez et al. 2025; Jongman et al. 2023; Payne and Federmeier 2017). These potential mechanisms may also be generally compatible with revision‐based interpretations of the anterior positivity, such that incorporating unpredicted information into one's mental model when there is another active representation “requires the reprioritization of meaning‐based information” (Federmeier 2022, 19). Some work has also distinguished the anterior positivity to prediction violations from a frontal negativity to expected words (Lai et al. 2023; Wlotko et al. 2012), which may reflect ambiguity resolution processes associated with selecting the most probable among one of multiple possible pre‐activated representations (Federmeier 2022; Lee and Federmeier 2009).

Other frameworks situate late positivities as “providing strong signals that the statistical structure of the broader communicative environment has *changed*,” which support meaning recovery in the face of unexpected input (Kuperberg et al. 2020, 31, emphasis added). Here, the anterior positivity relates to successful situation model updating elicited when processing unpredicted information (Kuperberg et al. 2020, 25). Follow‐up work by Brothers et al. (2023) presented participants with sentence contexts that constrained to multiple completions (i.e., best completion/expected and second‐best completion). They did not find evidence that second‐best completions had a larger frontal positivity than the best completions. Therefore, they conclude that this situation model updating does not require suppression of more likely predicted lexical information.

Because this is an active area of research, there is some disagreement in the literature regarding what the anterior positivity functionally represents. The two leading accounts discussed above generally frame the anterior positivity as explicitly related to revision‐based processing of prediction violations (e.g., Lai et al. 2023; Payne and Federmeier 2017; Hubbard and Federmeier 2021; Federmeier 2022) or as driven by broader updating of a situation model (e.g., Kuperberg et al. 2020; Brothers et al. 2020, 2023). Nevertheless, both anterior positivity accounts suggest that some additional processing is engaged in highly constraining contexts when encountering plausible information that is unpredicted. The current design was not built to differentiate between these accounts, so we remain agnostic on this front. Our aims for this paper are to test whether frontally distributed responses are indeed detectable in naturalistic reading, where behavioral effects of encountering prediction violations (compared to merely unexpected input) have not been consistently found (which we discuss further in the next section).

In contrast to the anterior positivity, the LPC has been consistently linked to processing difficulties when readers encounter semantically anomalous or incongruent information, requiring additional processing to form a coherent representation (Brouwer et al. 2012). Some models explicitly link the N400 with the LPC as part of a single‐stream architecture, such that the N400 indexes early stages of semantic memory retrieval whereas the LPC indexes a continuous measure of integration effort of word meaning into the building sentence representation (Brouwer et al. 2017; Aurnhammer et al. 2023). Other models link the LPC more explicitly to specific stages of processing incongruent information. For example, Kuperberg et al. (2020) suggest that the LPC to semantic anomalies is triggered by high‐level detection of conflict between the input and the current state of one's situation model, which may be associated with downstream attempts at re‐analysis or repair (Kuperberg et al. 2020; see also Van Petten and Luka 2012; van de Meerendonk et al. 2010). Consistent with this latter interpretation, the magnitude of both syntactic P600 and semantic LPC effects is strongly associated with regressive eye movements, suggesting increased re‐processing behavior in natural reading (Metzner et al. 2017). Nevertheless, these accounts converge on predicting that the LPC is larger when encountering incongruous information. Therefore, understanding their role in normal reading is of high theoretical importance.

While these post‐N400 positivities have been explored and characterized extensively using single‐word RSVP, much less is known about how these processes may be elicited in naturalistic reading, for example, whether these components can be elicited in parafoveal vision, like the N400. Payne et al. (2019), using flanker RSVP, found no clear evidence for selective elicitation of the anterior positivity to unexpected words (see also Stites et al. 2017; Payne and Federmeier 2017). However, they did show evidence for an LPC elicited by foveal but not parafoveal viewing of the target (Payne et al. 2019). Notably, this effect was observed only when readers engaged in active comprehension via explicit plausibility judgments for each sentence, compared to passive reading, consistent with prior work suggesting the LPC is sensitive to task goals (e.g., Kolk et al. 2003). This foveally dependent LPC has been recently replicated by Milligan et al. (2023), who additionally showed that masking parafoveal viewing of the implausible word was not modulatory (further suggesting a foveal origin), and Li et al. (2023), who extended this finding to syntactic violations and the P600 as well. Collectively, this suggests that while early semantic retrieval may begin parafoveally, direct foveation is required to elicit later LPC effects. It remains unclear whether the anterior positivity to prediction violations can be elicited parafoveally or foveally.

### 1.2 Predictability and Plausibility Effects on Eye‐Movements

When compared to the ERP literature, eye‐tracking research has shown both converging and diverging evidence in context processing. Some studies have shown that young adults skip more often and spend less time reading predictable compared to unpredictable words (Rayner et al. 2006, 2011; see Staub 2015 for a review). While there are clear predictability benefits for processing contextually expected words, there has been mixed evidence of a specific cost to prediction violations from unexpected but plausible words, and sometimes readers may even show a processing benefit. For example, Luke and Christianson (2016) showed when lexical competitors are present (i.e., there is a more expected/higher cloze word), there were facilitative effects, rather than inhibitory costs in natural reading. To assess prediction costs, Frisson et al. (2017) compared fixation durations on unexpected targets in neutral contexts (e.g., “The widow thought that it was a lovely *garden*…”) and constraining contexts (e.g., “The priest wondered how he could get more people to come to the *garden*…”). They found classic predictability benefits for expected targets (e.g., “*church*…”) and semantically related unexpected targets (e.g., “*sermon*…”), but no evidence for predictability costs (i.e., slower reading times on unpredictable words). This divergence between eye movement data and some ERP studies on the anterior positivity led Frisson et al. (2017) to argue that the anterior positivity may be an artifact of unnatural RSVP presentation typical of ERP studies. However, casting doubt on the ubiquity of null findings for behavioral prediction costs, Wong, Veldre, and Andrews (2024) recently showed evidence for prediction costs in the eye‐movement record: when anomalies were not present, unrelated unpredictable targets disrupted reading on first fixation and gaze duration. They interpret lexical prediction as being sensitive to overall environmental demands, such that readers may have shifted to a more top‐down approach when only plausible sentences were presented. This finding contrasts with electrophysiological evidence showing additional processing of plausible prediction violations in the presence of other trials with semantic anomalies (DeLong et al. 2014; Quante et al. 2018; Federmeier et al. 2010; Ryskin et al. 2020). Finally, two studies have shown evidence of behavioral costs when encountering prediction violations in self‐paced reading (Payne and Federmeier 2017; Jongman et al. 2023).

In addition to predictability, readers are rapidly sensitive to plausibility, evident in plausibility preview effects in parafoveal vision, where skipping rate decreases and fixation time increases for implausible previews, independent of word frequency and length (Veldre et al. 2020). When plausibility violations *are* fixated, readers show early and large disruptions (Rayner et al. 2004; Warren and McConnell 2007; Warren et al. 2015). For instance, Rayner et al. (2004) showed that outright anomalous targets elicit an effect of plausibility in first‐pass reading (i.e., gaze duration). While eye‐movement data show plausibility violation disruptions onset quickly, ERP evidence additionally shows that these effects persist for hundreds of milliseconds, and some integrative effects may not be initiated until foveal viewing (Payne and Federmeier 2017; Payne et al. 2019; Milligan et al. 2022; C. Li et al. 2023).

### 1.3 Context Processing: Evidence From Co‐Registered EEG and Eye‐Movements

Co‐registration offers strong promise toward overcoming limitations with RSVP ERP reading methods, improving ecological validity and generalizability. Recent work on FRPs has shown that co‐registration can provide a nuanced measure of the time course of lexical and semantic effects in natural reading. Dimigen et al. (2011) investigated predictability during natural reading and found similar patterns to what has been found in independent ET and EEG studies: low‐predictability targets were fixated longer than high‐predictability targets in early measures including first fixation and gaze duration. They also showed a graded N400 effect as a function of predictability, and this component had a potentially earlier onset than in RSVP. Kretzschmar et al. (2015) showed similar results, such that more predictable words had fewer regressions, were skipped more often, and showed facilitated N400 effects, but they did not find evidence for parafoveal predictability effects, despite some earlier work showing a parafoveal N400 (Kretzschmar et al. 2009).

Metzner et al. (2015) showed that N400 responses to (semantically valid) world knowledge violations (e.g., *red* school buses) were similar in both RSVP and co‐registration designs. Interestingly, they later showed that natural reading with regressive eye movements improved comprehension compared to RSVP (Metzner et al. 2017). Regressive trials showed an LPC to semantic and syntactic violations. In contrast, trials without regressions showed poorer comprehension and a sustained centro‐parietal negativity for sentence‐final violations. They suggest this pattern reflects strategy differences contingent on prediction error detection for an incongruent word. However, no existing co‐registration studies have shown an anterior positivity to prediction violations. Overall, these studies highlight the benefits of natural reading, and the importance of allowing readers to volitionally interact with text.

### 1.4 The Current Study

We conducted an experiment recording simultaneous eye movements and fixation‐related potentials while participants read highly constraining sentences with expected, unexpected (but plausible), and anomalous target words. Thus, we adopted the same design as DeLong et al. (2014), allowing for a conceptual replication to investigate how semantic predictability and plausibility affect online sentence processing within an ecologically valid and naturalistic reading context. Critically, our aim was to simultaneously examine the N400 and post‐N400 positivities time locked to first fixations on both pre‐target and target words, the first study to do so in natural reading (to our knowledge). This affords an investigation into parafoveal and foveal lexical and semantic processing at the behavioral and electrophysiological level in natural reading. We expected to observe a semantically graded N400 effect when time locked to the pre‐target word, replicating and extending both flanker ERP and co‐registration findings. Critically, we expected the subsequent N400 time locked to the target word to be facilitated (i.e., attenuated) upon foveation. We also expected to observe robust plausibility effects (i.e., early sensitivity to semantic anomalies) in both ET and EEG measures, as well as in more delayed indices, including a foveal LPC to anomalous targets (replicating Metzner et al. 2017 and C. Li et al. 2023). We expected early predictability effects in both methods (e.g., facilitation in gaze duration and the N400, as described above), as well as later sensitivity to prediction violations in the FRP record, in the form of an anterior positivity to unexpected but plausible targets. A critical question concerns whether this effect, like the LPC in prior work, is elicited at fixation, or can be elicited beginning in parafoveal vision. Additionally, this study affords a test of prediction violations in both FRPs and eye movements simultaneously, which Wong, Reichle, and Veldre (2024) argue is necessary for theoretical development, allowing for a more direct bridge between these two literatures reporting conflicting results with respect to predictive processing.

## 2 Methods

This study was preregistered on the Open Science Foundation website (https://osf.io/f8t4z). All Supporting Information can be found here (https://osf.io/4t3xk/?view_only=341dc9492d6f4245a33b5ccc5ffa823c). As reported in the preregistration, sensitivity power analyses show that with a sample size of 48, we are powered at 0.80 to detect a standardized effect size of 0.32 with 162 experimental items (Westfall 2015). Unless specified as exploratory, all reported analyses were a priori. Any deviations from the preregistration are reported and justified.

### 2.1 Participants

50 young adults (*M* = 21, range: 18–32 years old, 34 female) from the University of Utah and Salt Lake City community participated for course credit or payment of $10 per hour. All subjects reported normal or corrected‐to‐normal vision (NA = 1) and reported English as their native language (NA = 1).^1^ Seven subjects were excluded due to not meeting inclusion criteria, technical errors during recording, and/or high missing data (> 60% in any condition). All subsequently reported analyses are from the remaining 43 subjects.^2^

### 2.2 Materials

Experimental stimuli consisted of 162 sentences with three within‐subject conditions. Each experimental item was a highly constraining sentence adapted from multiple sources, including Federmeier et al. (2007), Payne et al. (2016), and Peelle et al. (2020). Sentence contexts including the pre‐target word (and sentence endings) were held constant across conditions. Sentences differed at the target word depending on the expectancy condition such that the target was either expected, unexpected but plausible, or anomalous (following Delong et al. 2011, 2014; Payne et al. 2016). For example, “When the power went out the house became *dark/cold/half* like last time.” Pre‐target (n) words were at least 4 characters long to limit target (*n* + 1) foveal viewing (*M* = 5.83, range = 4–9 characters). Target words were at least 4 characters long to reduce skip rates (*M* = 4.96, range = 4–9 characters). Target words were lexically matched on frequency, length, orthographic neighborhood, imageability, and concreteness. Frequency was measured via the log‐transformed hyperspace analogue to language (HAL) and subtitle frequency (SUBTLWFus; Brysbaert and New 2009) measures, orthographic neighborhood was measured with the OLD20 measure (the average orthographic Levenshtein distance of the twenty closest neighbors; Yarkoni et al. 2008), concreteness ratings were selected from the English Lexicon Project database (Balota et al. 2007), and imageability ratings were selected from MRC Psycholinguistics Database (Wilson 1988). Norming data were also collected for cloze probability, plausibility ratings, and possibility ratings. Participants were recruited from Prolific and paid $10. For the cloze probability ratings, 15 subjects were recruited to provide the target word given the sentence context. For the plausibility and possibility ratings, 31 subjects judged sentence fragments including the target word, counterbalanced across 3 lists. Final stimulus set descriptive statistics for the target words are reported in Table 1. In the final stimulus set, all pairwise comparisons across expectancy conditions for lexically matched characteristics were non‐significant (*p* > 0.05), except for a small difference between expected and unexpected conditions for imageability (*p* = 0.04).

**TABLE 1:** Stimuli characteristics and norming results for target words.

Characteristic	Expected	Unexpected	Anomalous
Length	4.99 (1.16)	4.94 (1.08)	4.94 (0.98)
Log HAL	9.66 (1.57)	9.64 (1.54)	9.79 (1.39)
Log SUBTLWF	3.31 (0.74)	3.19 (0.68)	3.18 (0.70)
Orthographic neighborhood	1.68 (0.49)	1.69 (0.41)	1.71 (0.40)
Concreteness	4.06 (0.87)	3.94 (0.90)	3.97 (0.98)
Imageability	527.59 (86.26)	498.67 (83.61)	511.45 (89.55)
Cloze probability	0.74 (0.24)	0.03 (0.08)	0 (0.0)
Plausibility ratings	4.53 (0.46)	3.99 (0.72)	1.9 (0.59)
Possibility ratings	3.69 (0.34)	3.32 (0.51)	1.73 (0.47)

*Note:* Means are reported with standard deviations in parentheses. Plausibility ratings are on a scale of 1 (not at all plausible) to 5 (entirely plausible). Possibility ratings are on a scale of 1 (impossible) to 4 (possible).

### 2.3 Procedure

In a dim, quiet room, participants were seated 80 cm from the presentation monitor (ASUS VG248 LCD) with their heads stabilized in a mounted chin rest. Electrophysiological and eye‐movement responses were recorded simultaneously while participants read sentences silently. Screen resolution was set to 1920 (W) x 1080 (H) with 32 bits per pixel and a refresh rate of 60 Hz. Text was presented in Courier New size 18 font (14 pixels per character), which is about 3.5 characters per degree visual angle. All text was presented on a single line. The experiment was programmed in Experiment Builder (SR Research). Real‐time event markers (i.e., Biometric TTL pulses) were sent when readers fixated on critical regions of interest using a fixation‐contingent trigger. Additionally, experiment and item‐level event markers were shared across the EEG and ET data streams using a combination of TTL parallel port markers and ASCII text messages delivered via ethernet cable. Participants were instructed to read sentences as they normally would for comprehension. The sentence remained on the screen for 1000 ms after keypress. After each trial, participants made a two‐alternative forced choice plausibility judgment by responding to the prompt: “Plausible?” with either yes or no. This served not only as a basic attention check but also introduced an online task. This is important because we were interested in post‐N400 semantic effects and prior work (e.g., Payne et al. 2019) shows using explicit plausibility judgments elicits larger LPC effects to anomalous targets. Participants were permitted to take breaks as needed and explicitly notified when they were halfway through.

### 2.4 Electroencephalography (EEG) and Eye‐Movement Recording

EEG was recorded from 32 silver‐silver chloride actiCAP slim active electrodes distributed by Brain Products. Electrodes were spaced according to the international 10–20 localization system for 32 channels. Electrodes were referenced online to the TP10 electrode, and re‐referenced offline to the average of the TP10 and TP9 electrodes, which are close to the right and left mastoids, respectively. Electrode impedances were kept below 20 kΩ. Continuous EEG was amplified through an actiCHamp amplifier (connected to actiPOWER battery) with an online highpass filter of 1000 Hz and recorded at a rate of 500 Hz using BrainVision Recorder software. Eye movements were continuously recorded at a rate of 1000 Hz from the right eye during each trial with an Eyelink 1000 Plus desktop‐mounted infrared eye‐tracker camera. Participants completed a 9‐point calibration prior to the start of the experiment. Additionally, before each trial, there was a left‐aligned drift check followed by a black square gazebox aligned to the sentence starting position triggered automatically by direct fixation.

### 2.5 Data Processing

#### 2.5.1 Synchronizing Data Streams

Co‐registration data processing followed recommendations by Dimigen et al. (2011) via the EYE‐EEG toolbox and those made by Degno et al. (2021). Raw continuous eye‐tracking data and EEG recordings were parsed and synchronized using the EYE‐EEG toolbox based on shared start and end event latencies across the data streams. For each subject, synchronization quality was assessed by examining the number of shared events across data streams (up to 832/subject) as well as through visual inspection of the synchronization plot and data scroll. For pilot and initial subjects, synchronization accuracy was additionally assessed using a cross‐correlation function (in the EYE‐EEG toolbox) between the monopolar left and right horizontal EOG channels and ET horizontal gaze position.

#### 2.5.2 Electrophysiology Processing

Synchronized continuous EEG data were filtered with a bandpass filter of 0.1–100 Hz using EEGLAB. Periods of continuous EEG with blinks or out‐of‐range data (i.e., outside of 1920 × 1080 pixels) in the eye‐movement record were removed via the EYE‐EEG toolbox *pop_rej_eyecontin()* function. A peak‐to‐peak moving window algorithm in ERPLAB was then applied across the continuous data to remove any additional large artifacts (i.e., Commonly Recorded Artifactual Potentials; Luck 2022). Remaining ocular artifacts were removed using an eye‐movement‐guided, optimized independent component analysis procedure (OPTICAT; Dimigen 2020; see Materials S1 for more details). Standard ERP processing was then conducted using EEGLAB and ERPLAB. Data were down‐sampled to 250 Hz. EEG channels were low‐pass filtered at 30 Hz. Epoched EEG data (time locked to target word and pre‐target word fixation) were examined for any remaining artifacts, including eyeblinks, line noise, signal drift, and eye movements. Fixed thresholds for each artifact detection algorithm were set and then validated through a condition‐blind visual inspection of the raw data, following our standard lab procedures as reported elsewhere (e.g., Payne et al. 2015; Payne and Federmeier 2017, 2018; Silcox and Payne 2021). Any trials flagged as containing residual artifacts were excluded from further analyses.

#### 2.5.3 Eye‐Movement Processing

Eye‐tracking data were processed using Data Viewer by SR Research. All raw EDF files were imported and practice trials were removed. Sentence periods were created starting after the gaze box (when the sentence appears) and ended at a subject keypress indicating they were finished reading. Each trial was visually inspected for extreme drift. Standard fixation cleaning was conducted, whereby fixations shorter than 80 ms and within 1 character of another fixation were merged and single fixation durations that exceeded 800 ms and those shorter than 80 ms were excluded.

### 2.6 Data Analyses

#### 2.6.1 Fixation‐Related Potential Regions of Interest (ROIs) and Analyses

For both the pre‐target and target words, we calculated mean amplitudes for pre‐specified spatiotemporal ROIs. For the N400, we measured mean amplitude from 300 to 500 ms after fixation onset at six centro‐posterior electrode sites (CP1, CP2, Cz, P3, P4, and Pz). For the LPC, we measured mean amplitude from 600 to 800 ms after fixation onset at six posterior electrode sites (P3, Pz, P4, O1, Oz, and O2).^3^ For the anterior positivity, we measured mean amplitude from 600 to 800 ms after fixation onset at seven frontal sites (FP1, FP2, F7, F3, Fz, F4, and F8). Separate linear mixed effect models were fit for each outcome measure using the *lme4* (Bates et al. 2015) and *afex* (Singmann et al. 2017) packages in R. The fixed effect of context has three levels: expected, unexpected, and anomalous. We report regression coefficients, and show the effect size in milliseconds, standard errors, and omnibus likelihood ratio tests. Random effects included a random intercept for participants (but not items, since FRPs were first aggregated across items following traditional ERP methods), and we defined random slopes across participants using the maximal random effects structure that was supported by the data (see Barr et al. 2013; Bates et al. 2015). When a maximal model failed to converge, we used a backward selection procedure to reduce model complexity (Barr et al. 2013). After fitting each linear mixed model, we conducted post hoc analyses using the *emmeans* package in R (Lenth 2024). Degrees of freedom were estimated using the Kenward–Roger method. Following DeLong et al. (2014), who had a similar design, we tested contrasts between each of the three conditions: (1) Expected versus Unexpected, (2) Expected versus Anomalous, and (3) Unexpected versus Anomalous. We controlled for multiple pair‐wise comparisons via the Bonferroni method.^4^

#### 2.6.2 Exploratory Mass Univariate FRP Analyses

We additionally conducted cluster‐based permutation tests^5^ (Maris and Oostenveld 2007) based on the cluster mass statistic (Bullmore et al. 1999) using a family‐wise alpha of 0.05. We used constrained spatiotemporal ROIs for improved power with only theoretically relevant pairwise contrasts to reduce Type 1 error rates while maintaining high statistical power (Fields and Kuperberg 2020). For the N400, a constrained spatiotemporal ROI of 300–500 ms (101 time points) at 6 centro‐parietal scalp electrodes (CP1, CP2, Cz, P3, P4, Pz) was included for each contrast test (i.e., 606 comparisons). For the N400, we tested each possible pairwise contrast for both pre‐target and target regions. For the anterior positivity, a constrained spatiotemporal ROI of 600–1000 ms (201 time points) at 7 frontal electrodes (FP1, FP2, F3, F7, Fz, F4, F8) was included for each contrast test (i.e., 1407 comparisons). For the anterior positivity, we tested U vs. E and U vs. A contrasts at pre‐target and target regions. For the LPC, a constrained spatiotemporal ROI of 600–1000 ms (201 time points) at 6 posterior electrodes (P3, Pz, P4, O1, Oz, O2) was included for each contrast test (i.e., 1206 comparisons). For the LPC, we tested A vs. U contrasts at both pre‐target and target regions. Any electrodes within approximately 5.44 cm of one another were considered spatial neighbors. Repeated measure t‐tests were performed for each comparison using the original data and 2500 random within‐participant permutations of the data. For each permutation, all t‐scores corresponding to uncorrected *p*‐values of 0.05 or less were formed into clusters. The sum of t‐scores in each cluster is the “mass” of that cluster and the most extreme cluster mass in each of the 2501 sets of tests was recorded and used to estimate the distribution of the null hypothesis. These analyses were conducted via the Mass Univariate ERP Toolbox (Groppe et al. 2011).

#### 2.6.3 Eye‐Movement Analyses

We analyzed the following ET measures: single fixation duration, first fixation duration, probability of skipping, probability of re‐fixating, gaze duration, regression path duration, probability of regressing out of a region, and total reading time.^6^ Linear mixed effects models were fit to the duration‐based measures and logit mixed models to the probability‐based eye movement measures. For duration‐based measures, we report models on raw scale (ms). The fixed effect of context has three levels: expected, unexpected, and anomalous. We report regression coefficients, which show the effect size in milliseconds, standard errors, and likelihood ratio tests. Random effects included crossed random intercepts for both participants and items, and we defined random slope variance and covariance terms using the maximal random effects structure supported by the data (see Barr et al. 2013; Bates et al. 2015). When a maximal model failed to converge, we used a backward selection procedure to reduce model complexity (Barr et al. 2013). As described in the EEG section, the same procedure was followed for pairwise contrasts across conditions.

## 3 Results

### 3.1 Online Plausibility Judgment Results

Online plausibility judgments made after each trial encouraged participants to engage in *active* comprehension. Performance was high on these plausibility judgments with average accuracy at 89.8% (SD = 0.30) across all subjects and conditions, suggesting that readers were able to successfully discriminate between plausible and implausible sentences. Reported FRP and eye‐movement analyses are based on all trials (including both incorrect and correct judgments).

### 3.2 Fixation‐Related Potential Results

**FIGURE 1:**
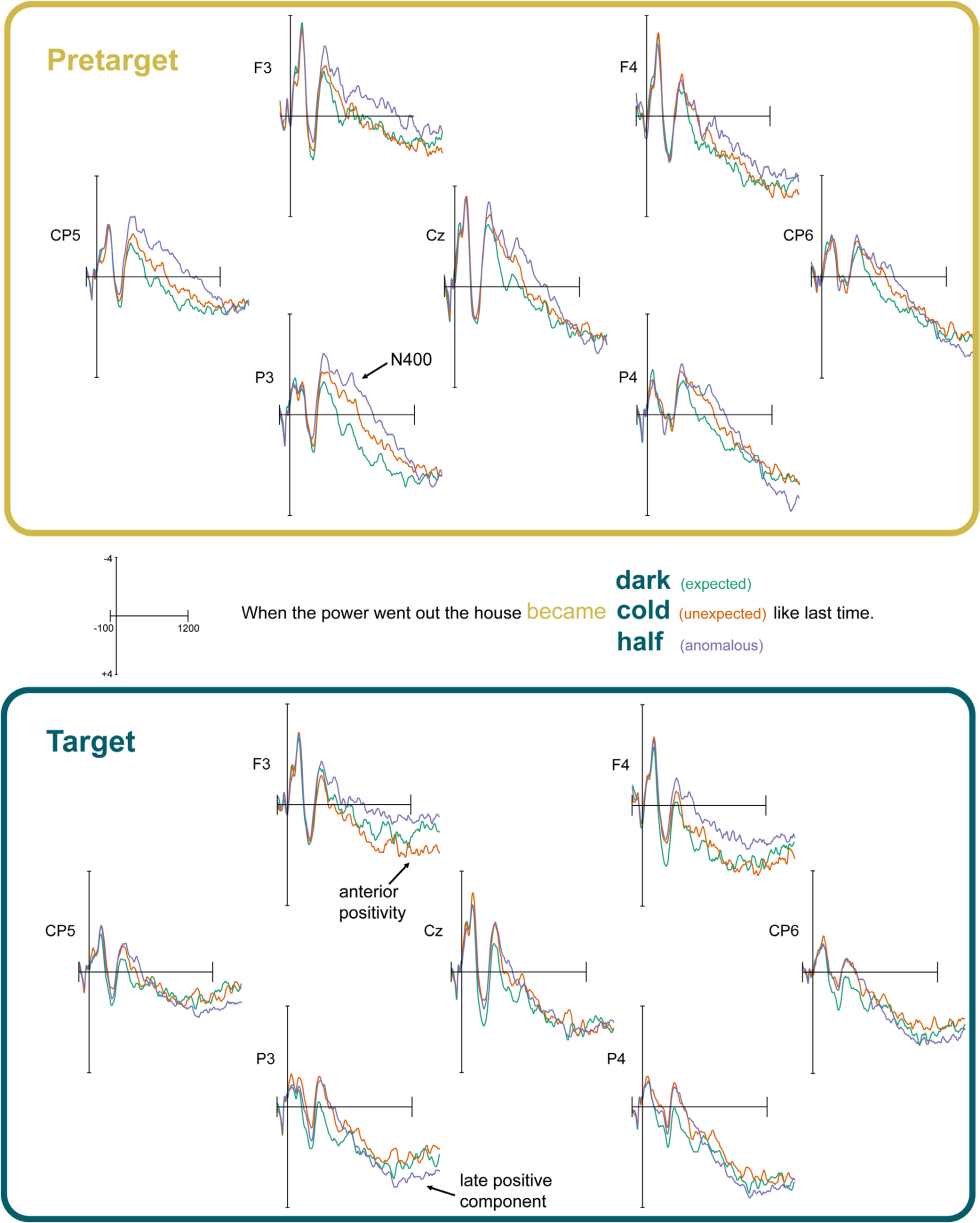
Selected frontal, parietal, and posterior electrodes for pre‐target and target regions of interest.

We report analyses conducted for three FRP components at both pre‐target and target fixations, which include an N400, anterior positivity, and LPC. In each respective section, we first report the analyses using standard ERP methods with a priori spatiotemporal regions of interest.^7^ We also report cluster‐based permutation tests on theoretically relevant condition contrasts and spatio‐temporal ROIs (Fields and Kuperberg 2020).^8^ Figure 1 shows representative electrodes across the scalp at pre‐target and target fixations.

#### 3.2.1 N400: Do Readers Access Semantic Information Prior to Foveal Target Fixation in Natural Reading?

We were interested in evaluating whether readers begin extracting contextual information before foveal viewing of target words, leading to a large parafoveal N400 effect, and whether this parafoveal activation would subsequently facilitate semantic memory retrieval at direct target fixation, leading to a reduced foveal N400 effect, as previously observed in the flanker paradigm (e.g., Payne et al. 2019).

##### 3.2.1.1 Parafoveal Effects

Are readers uniquely sensitive to semantic expectancy and plausibility information parafoveally? Indeed, we found that when time‐locked to the pre‐target word (parafoveal viewing of target varying by contextual condition), there was a significant main effect of target word condition on the N400, an index of semantic access (*χ* ^2^ (2) = 16.63, *p* < 0.001). Follow‐up pairwise contrasts between the estimated marginal means showed a significant difference between anomalous and expected conditions, such that pre‐target words preceding anomalous target words were 1.32 μV more negative (*t* (88) = −4.10, *p* = 0.0003). Pre‐target words preceded by unexpected target words were 0.95 μV more negative than expected targets (*t* (88) = 2.95, *p* = 0.012). No significant difference was observed between anomalous and unexpected target words (*t* (88) = −1.15, *p* = 0.75).

**FIGURE 2:**
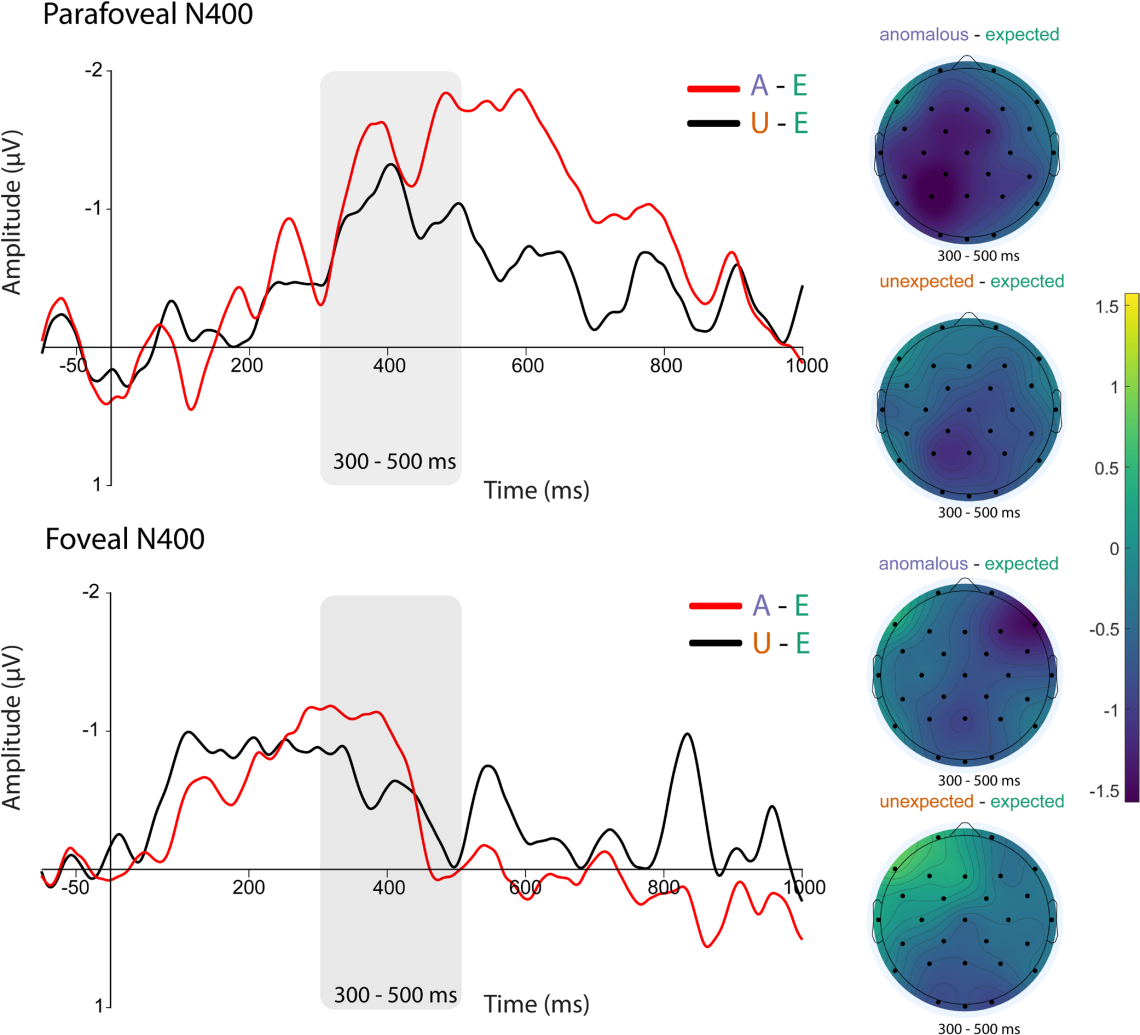
N400 context effects time‐locked to pre‐target fixation (top) and target fixation (bottom). (Top) Difference waves and scalp topographies for significant condition contrasts time‐locked to first fixation in the pre‐target region. (Bottom) Comparison figures for FRPs time‐locked to first fixation at the target word. We applied a low‐pass filter of 15 Hz on difference waves for data visualization purposes only. Scalp topographies corresponding to mean amplitude between 300 and 500 ms are plotted for each contrast to the right of the waveforms for both pre‐target and target FRPs.

Consistent with these a priori analyses at the pre‐target region, there was a significant negative cluster for the anomalous condition compared to the expected condition across nearly the entire region in the mass univariate analysis. There was also one significant negative cluster for the unexpected compared to expected condition approximately 320–500 ms at all included electrodes (although this is slightly less central). There were no significant clusters for the anomalous versus unexpected contrast. These results provide evidence consistent with a parafoveally driven N400 context effect, where readers are able to extract expectancy and plausibility information when fixating the pre‐target word. Figure 2 (top) shows the difference waves for these significant contrasts, as well as the scalp topographies in this 300–500 ms time‐window.

##### 3.2.1.2 Foveal Effects

We were also interested in whether readers would still elicit an N400 at the target word if they had already started retrieving respective semantic information from long‐term memory parafoveally. We found that time‐locked to the target word, there was a significant main effect of target word condition (*χ* ^2^ (2) = 6.84, *p* = 0.033) on the N400. Pairwise contrasts between the estimated marginal means showed that anomalous target words were 0.75 μV more negative in mean amplitude compared to expected targets (*t* (88) = −2.5, *p* = 0.038). There were no significant differences between anomalous and unexpected target words (*t* (88) = −0.66, *p* = 1.0), nor between expected and unexpected targets (*t* (88) = 1.88, *p* = 0.19), suggestive of a weaker N400 response at the target word.

Consistent with these a priori analyses, there was one significant negative cluster for the anomalous targets compared to expected approximately 300–430 ms at all included electrodes in the mass univariate analysis. There were no other significant clusters at the target. These results suggest that there is some continued N400 activation at foveal viewing. Figure 2 (bottom) illustrates this reduced N400 in the same difference waves and scalp topographies as the pre‐target region. This same comparison is also visible in Figure 1 if you compare P3 across pre‐target and target regions.

#### 3.2.2 Anterior Positivity: Are Readers Sensitive to Prediction Violations in Natural Reading?

**FIGURE 3:**
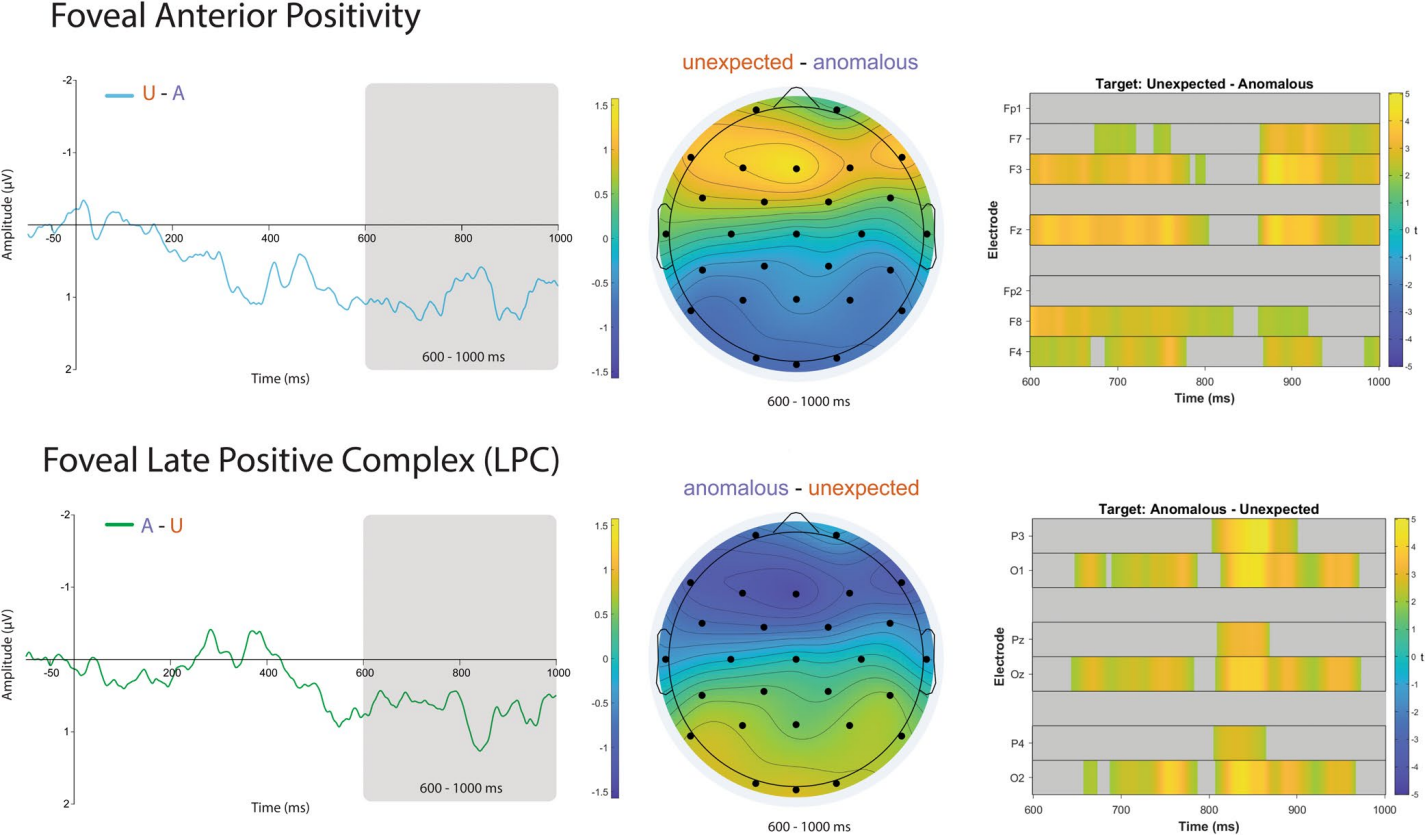
Late positivities at target fixation. Top: (Left) Anterior positivity difference wave (unexpected—anomalous) grand averaged across frontal electrodes (Fp1, F7, F3, Fz, Fp2, F8, F4). (Middle) Scalp topography for mean amplitude across 600–1000 ms for unexpected minus anomalous contrast. (Right) Constrained mass univariate raster plot for frontal electrodes from 600 to 1000 ms. Bottom: (Left) Late positive component difference wave (anomalous—unexpected) grand averaged across posterior electrodes (P3, O1, Pz, Oz, P4, O2). (Middle) Scalp topography for mean amplitude across 600–1000 ms for anomalous minus unexpected contrast. (Right) Restricted mass univariate raster plot for posterior electrodes from 600 to 1000 ms. Difference waves were low‐pass filtered at 15 Hz.

We were interested in whether we would observe evidence for frontally distributed late positive activation to unexpected but plausible target words, an effect that has been linked to the detection and processing of prediction violations (e.g., Lai et al. 2023; Federmeier 2022). At the target word, we expected to observe a late anterior positivity to unexpected but plausible targets, where the reader may have to overcome a prediction failure and/or update their situational representation in response to unexpected input. Figure 3 shows the difference waves, scalp topographies, and constrained raster plots for the two significant contrasts most relevant to understanding the late positive effects.

##### 3.2.2.1 Parafoveal Effects

Just like the N400, we tested whether the anterior positivity was elicited parafoveally by first time‐locking to the pre‐target word. At the pre‐target word, there was a significant main effect of word condition (*χ* ^2^ (2) = 6.79, *p* = 0.03). However, pairwise contrasts between the estimated marginal means showed that there were only marginal differences between anomalous and expected conditions (*t* (88) = −2.36, *p* = 0.06) and anomalous and unexpected conditions (*t* (88) = −2.19, *p* = 0.09), and there was not a significant difference between expected and unexpected conditions (*t* (88) = −0.17, *p* = 1.0).

For the mass univariate analyses with an extended time‐window of 600–1000 ms, the unexpected minus anomalous contrast had a significant positive cluster from approximately 678–868 in Fz and F3. This cluster likely reflects volume conduction of the continued distributed parafoveal N400 activation (rather than a genuine parafoveal frontal positivity), as seen in Figure 1.^9^ There were no significant clusters for the unexpected vs. expected contrast. Taken together, these results suggest the anterior positivity cannot be reliably observed parafoveally, consistent with prior ERP work in the flanker paradigm (Stites et al. 2017; Payne et al. 2019).

##### 3.2.2.2 Foveal Effects

Consistent with our predictions, time‐locked to the target word, there was a significant main effect of target word condition (*χ* ^2^ (2) = 12.61, *p* < 0.01). Follow‐up pairwise contrasts showed there was a marginally significant difference between the anomalous and expected targets (*t* (88) = −2.17, *p* = 0.10). Importantly, there was a significant difference between the anomalous and unexpected targets, such that unexpected target words were 1.08 μV more positive than the anomalous targets (*t* (88) = −3.62, *p* < 0.01), consistent with an anterior positivity to prediction violations. The contrast between expected and unexpected was not significant (*t* (88) = −1.45, *p* = 0.45). Consistent with a foveal anterior positivity, mass univariate analyses showed two positive clusters to unexpected targets compared to anomalous targets across approximately the entire ROI (except for 832 to 862 ms and Fp1 and Fp2 electrodes, see Figure 3 top‐right). These results provide clear evidence for a foveally driven response to unexpected targets in high‐constraint contexts. There were no significant clusters for the unexpected vs. expected contrast.

#### 3.2.3 LPC: Are Readers Sensitive to Semantic Anomalies in Natural Reading?

We were interested in whether and when readers would show increased activation when encountering semantically anomalous words. At the target word, we expected a posteriorly distributed late positive component (LPC) to foveal viewing of anomalous targets, likely an index of the failure to integrate an anomalous word.

##### 3.2.3.1 Parafoveal Effects

Just like the other FRPs, we tested whether readers initiated LPC activation parafoveally. We found that time‐locked to the pre‐target word, there was a significant main effect of target word condition (*χ* ^2^ (2) = 11.04, *p* < 0.01). Pairwise contrasts showed that there were significant differences between anomalous and expected conditions (*t* (88) = −3.33, *p* = 0.004), such that expected items were 0.89 μV more positive over posterior electrodes compared to anomalous conditions. However, this effect is in the *opposite* direction of the canonical LPC, and may reflect a continued parafoveal N400 effect, as discussed below (see also Figure 1). There were no significant contrasts between anomalous and unexpected conditions (*t* (88) = −1.09, *p* = 0.84), but the expected condition was trending towards being more positive than the unexpected condition (*t* (88) = 2.24, *p* = 0.08), again consistent with a sustained N400 effect. Additionally with an extended time‐window of 600–1000 ms, mass univariate analyses show there was one significant positive cluster approximately 912 to 1000 ms at O1, O2, and Oz for the anomalous minus unexpected contrast in the direction of the LPC (see P4 in Figure 1). Because of its onset near the end of the epoch, it is likely not parafoveal in origin.^10^

##### 3.2.3.2 Foveal Effects

At direct fixation, time‐locked to the target word, there was a significant main effect of target word condition (*χ* ^2^ (2) = 7.55, *p* = 0.023) in the LPC spatiotemporal region. Pairwise contrasts showed that there were no significant differences between anomalous and expected targets (*t* (88) = −0.08, *p* = 1.0). However, consistent with the canonical LPC, anomalous targets were 0.58 μV more positive than unexpected targets (*t* (88) = 2.44, *p* = 0.0499). Expected targets were trending towards being more positive than unexpected targets (*t* (88) = 2.37, *p* = 0.061). Consistent with the a priori analyses, mass univariate analyses with an extended time‐window of 600–1000 ms showed two significant positive clusters to the anomalous condition compared to the unexpected condition (see Figure 3, bottom‐right). These clusters were approximately 644–786 ms at O1, O2, and Oz (possibly the same pre‐target cluster reported above) and from 804 to 972 ms at all included electrodes. These results suggest a selective posterior positivity to anomalous targets.

### 3.3 Eye‐Movement Results

**FIGURE 4:**
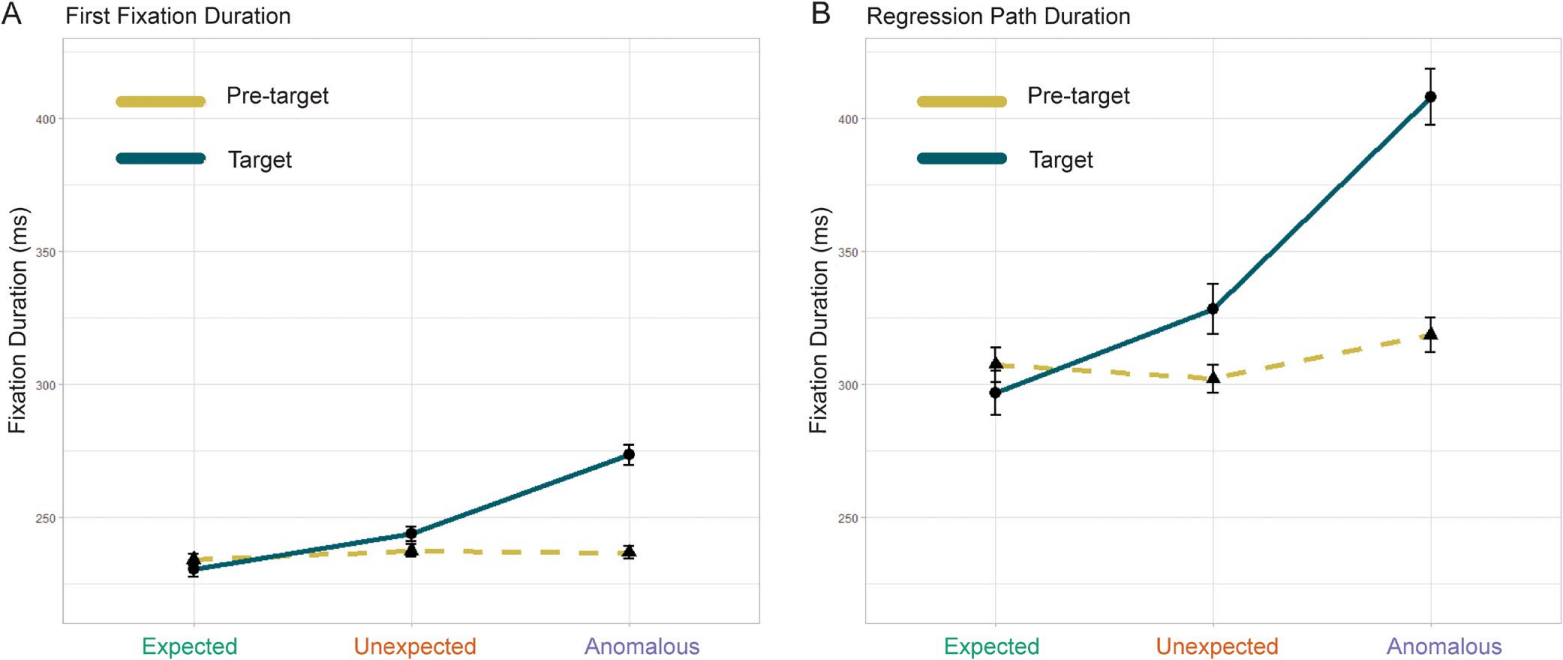
Representative eye‐tracking reading measures. Means and standard errors by condition and word region. Readers do not show predictability or plausibility effects at the pre‐target word, only at the target word. (A) First fixation duration (FFD) and (B) regression path duration (RPD). Pre‐target means are plotted as triangles and target means are plotted as circles. Dashed lines connect pre‐target means, while solid lines connect target means. Error bars are within‐subject. All target contrasts are significant for FFD and RPD. Scales are matched across the two plots.

We have selectively reported eye‐tracking outcomes below, including indices of early word recognition, re‐reading, and cumulative processing. Table 2 reports means and standard errors for representative reading measures by region of interest and condition.^11^ Figure 4 shows means and standard errors for two representative eye‐tracking measures: first fixation duration and regression path duration.^12^

**TABLE 2:** Means (and standard errors) for selected reading measures.

	Pre‐target			Target		
	Anomalous	Unexpected	Expected	Anomalous	Unexpected	Expected
*Duration‐based measures*						
First fixation duration	237 (2)	237 (2)	234 (2)	271 (3)	242 (3)	228 (3)
Gaze duration	262 (3)	257 (3)	257 (3)	300 (4)	257 (3)	239 (3)
Regression path duration	319 (6)	302 (5)	307 (6)	403 (10)	321 (8)	289 (8)
*Probability‐based measures*						
Probability of skip	0.33 (0.01)	0.33 (0.01)	0.33 (0.01)	0.45 (0.01)	0.46 (0.01)	0.49 (0.01)
Probability of regressing	0.11 (0.01)	0.10 (0.01)	0.10 (0.01)	0.12 (0.01)	0.09 (0.01)	0.07 (0.01)
Probability of re‐fixating	0.11 (0.01)	0.09 (0.01)	0.10 (0.01)	0.10 (0.01)	0.06 (0.01)	0.05 (0.01)

*Note:* Values reported by condition for pre‐target and target regions of interest. Values are reported in milliseconds for duration‐based measures and probabilities for probability‐based measures.

#### 3.3.1 Initial Word Recognition Measures

A primary goal in this study was to evaluate within the same sample and items whether we would observe evidence for prediction *costs* to unexpected targets. Prior work has shown differential evidence for the existence of costs to mis‐predicting in highly constraining contexts depending on the methodology (see Staub 2025 for a recent review). Specifically, most eye‐tracking research (but cf. Wong, Veldre, and Andrews 2024) does not show any behavioral costs to receiving unexpected targets compared to more expected completions. In general, we expected that we would observe evidence for predictability *benefits* in early eye‐tracking outcomes, such as shorter gaze durations and higher skip rates for expected targets. We hypothesized that we would not observe evidence for prediction *costs* to unexpected targets in early reading measures. The measures reported in this section are typically associated with early word recognition.

Consistent with the word regions analyzed in the FRP outcomes, we evaluated whether readers would show evidence for differential early parafoveal word recognition at the pre‐target word. There were no significant main effects of condition for first fixation duration (*χ* ^2^ (2) = 2.14, *p* = 0.34) or gaze duration (*χ* ^2^ (2) = 3.07, *p* = 0.22). While we did not see early predictability effects at the pre‐target region behaviorally, at the target word, first‐pass probability of skipping showed a significant main effect of condition (*χ* ^2^ (2) = 7.85, *p* = 0.02). Pairwise contrasts showed that expected targets (*EMM* = −0.03) multiplied the odds of skipping by 0.17 compared to anomalous targets (*EMM* = −0.19; *z* = −2.70, *p* = 0.02). The contrasts between anomalous and unexpected targets (*EMM* = −0.15; *z* = −0.70, *p* = 1.0), and between expected and unexpected targets were not significant (*z* = 2.00, *p* = 0.14). Because skip decisions are made before the word is fixated, a skip effect at the target word indicates a clear parafoveal effect.

Another aim of this study was to evaluate behavioral indices for encountering anomalous information, and when the presence of semantic anomalies exerts its influence. Unexpected vs. expected contrasts primarily reflect *predictability* effects, while unexpected vs. anomalous condition contrasts primarily reflect *plausibility* effects (E vs. A likely reflect predictability benefits *and* plausibility disruptions). Consistent with prior work, at the target word, for first fixation duration, there was a significant main effect of condition (*χ* ^2^ (2) = 161.68 *p* < 0.001). Pairwise contrasts showed that anomalous targets (*EMM* = 270 ms) were fixated for 43 ms longer than expected targets (*EMM* = 227; *t* (3440) = 12.56, *p* < 0.0001) and 29 ms longer than unexpected targets (*EMM* = 242 ms; *t* (3438) = 8.54, *p* < 0.0001). Unexpected targets were fixated for 14 ms longer than expected targets (*t* (3441) = −4.13, *p* = 0.0001). For gaze duration at the target word, there was a significant main effect of condition (*χ* ^2^ (2) = 205.42, *p* < 0.001). Pairwise contrasts showed that anomalous targets (*EMM* = 298 ms) were fixated for 61 ms longer than expected targets (*EMM* = 238; *t* (3436) = 14.04, *p* < 0.0001) and 43 ms longer than unexpected targets (*EMM* = 255 ms; *t* (3434) = 10.15, *p* < 0.0001). Unexpected targets were fixated for 18 ms longer than expected targets (*t* (3437) = −4.03, *p* < 0.001).

#### 3.3.2 Post‐Lexical Integrative Measures

The measures reported in this section are typically associated with disruptions triggered by post‐lexical integrative processing (e.g., Reichle et al. 2009). This formulation fits with models that allow for an interaction between oculomotor and parsing systems (for a discussion see Vasishth et al. 2013), such as anomalous information triggering a regression. We have also included in this section the cumulative eye‐tracking outcome of regression path duration, where we expected to primarily observe sensitivity to plausibility (e.g., Payne et al. 2016).

At the target word, the first‐pass probability of re‐fixation showed a significant main effect of condition (*χ* ^2^ (2) = 45.94, *p* < 0.001). Pairwise contrasts showed that anomalous targets (*EMM* = −2.42) multiplied the odds of re‐fixation by 0.85 compared to expected targets (*EMM* = −3.26; *z* = 6.35, *p* < 0.0001) and by 0.58 compared to unexpected targets (*EMM* = −3.00; *z* = 4.82, *p* < 0.0001). The expected versus unexpected contrast was not significant (*z* = −1.81, *p* = 0.21). At the target word, the first‐pass probability of regressing out of a region showed a significant main effect of condition (*χ* ^2^ (2) = 24.52, *p* < 0.001). Pairwise contrasts showed that anomalous targets (*EMM* = −2.23) multiplied the odds of regressing by 0.63 compared to expected targets (*EMM* = −2.85; *z* = 4.93, *p* < 0.0001) and 0.41 compared to unexpected targets (*EMM* = −2.64; *z* = 3.48, *p* = 0.0015). The expected versus unexpected contrast was not significant (*z* = −1.57, *p* = 0.35).

For regression path duration at the target word, there was a significant main effect of condition (*χ* ^2^ (2) = 139.17, *p* < 0.001). Pairwise contrasts showed that anomalous targets (*EMM* = 403 ms) were fixated for 116 ms longer than expected targets (*EMM* = 287; *t* (3432) = 11.51, *p* < 0.0001) and 83 ms longer than unexpected targets (*EMM* = 320 ms; *t* (3430) = 8.32, *p* < 0.0001). Unexpected targets were fixated for 33 ms longer than expected targets (*t* (3433) = −3.30, *p* < 0.01).

## 4 Discussion

This study investigated lexical and semantic processing across the visual field in natural reading via the co‐registration of eye movements and EEG. Co‐registration paradigms provide readers with the opportunity to interact with the text continuously through refixations, regressions, and re‐reading—a nuanced orchestration between our saccade planning and language processing systems. In the eye movement record, we largely replicated past work showing evidence of early and robust sensitivity to plausibility and expectancy at target word fixation, but limited evidence of parafoveal‐on‐foveal semantic influences. In contrast, when time‐locking first fixations to the pre‐target word, we observed a clear contextually graded parafoveal N400 effect: the N400 was largest in amplitude to anomalous parafoveal targets, followed by unexpected targets, and then expected targets. Strikingly, the N400 effects were *larger* in magnitude when time‐locked to the pre‐target word compared to time‐locked to the target word, consistent with past work using fixed‐gaze paradigms reporting foveal facilitation of the N400 following valid parafoveal preview (e.g., Milligan et al. 2023; Payne et al. 2019; Stites et al. 2017). At target fixation, we observed a late anteriorly distributed positivity to unexpected targets compared to anomalous targets, an effect that was not clearly present at the pre‐target word. Finally, we observed evidence for a posterior LPC to anomalous targets. While prior fixed‐gaze ERP paradigms have reported post‐N400 anterior sustained effects (e.g., DeLong et al. 2014; DeLong and Kutas 2020; Federmeier et al. 2007; Kuperberg et al. 2020), we believe this study is the first to replicate an anterior positivity to unexpected words in naturalistic sentence reading. In what follows, we discuss the implications of these findings for theories of semantic processing across the visual field in natural reading. We first discuss eye movement findings followed by FRPs. The discussion is generally structured such that, for each primary section of outcomes, we start by providing an independent discussion of the respective effect (e.g., parafoveal N400), discuss how it relates to other outcomes within the literature (e.g., related to flanker ERP studies), and then discuss cross‐literature implications (e.g., how FRPs inform our understanding of the relationship between lexical processing and oculomotor planning systems in reading).

### 4.1 Context Sensitivity in the Eye‐Movement Record

#### 4.1.1 Expectancy‐Based Facilitation in the Eye‐Movement Record

Our eye‐tracking results showed little evidence for early (i.e., at the pre‐target word) parafoveal sensitivity to expectancy, consistent with most ET research. The only statistically significant parafoveal effect we observed was expectancy‐based target skip decisions (the anomalous vs. expected contrast). Many studies have shown that predictable target words are skipped more often than unpredictable words (e.g., see Brysbaert et al. 2005; Staub 2015 for reviews). This target skipping effect is likely concurrently driven by decreased skipping of the anomalous targets, given prior work showing implausible previews are skipped less than plausible previews (Veldre et al. 2020). Since the target is visible parafoveally when skip decisions are made, they are thought to be initiated based on incomplete, or partial, parafoveal identification (Schotter 2018). In contrast to the pre‐target word, we see clear evidence for expectancy effects at the target word in early eye‐movement measures (i.e., first fixation duration and gaze duration), aligned with prior work. Staub (2015) argues that such effects reflect a reader's graded activation of lexical information such that readers pre‐activate a range of possible candidates, an effect that is facilitative for early lexical and/or prelexical processing.

#### 4.1.2 Plausibility‐Based Disruptions in the Eye‐Movement Record

We also show robust evidence in the eye‐movement record that readers are sensitive to plausibility violations in both first‐pass measures and in more cumulative duration‐based measures. Readers were immediately disrupted (i.e., upon target fixation) by semantic anomalies. Readers also had longer regression path durations on the anomalous target words compared to both unexpected and expected target words, suggesting that readers engaged in greater re‐processing of the context when encountering semantic anomalies, perhaps attempting to repair the incongruity. This replicates prior work showing that plausibility manipulations affect both immediate and downstream integrative processes (e.g., Rayner et al. 2004; Warren and McConnell 2007). These plausibility effects may reflect post‐lexical processes (Abbott and Staub 2015), including early detection of integration failure (Veldre et al. 2020).

#### 4.1.3 Accounting for Parafoveal Preview

However, since readers are receiving parafoveal information before fixating on the target words, target fixation durations likely do reflect preview information to some degree. Risse and Kliegl (2012) used the boundary change paradigm to show that previews of *n* + 2 words can elicit so‐called *delayed* parafoveal‐on‐foveal effects. That is, invalid information that was presented in parafoveal vision can have spillover effects that originate from parafoveal processing but appear in the eye‐movement record at a delay—after the eyes have already moved on to another word. Other accounts of parafoveal preview posit that at least some target fixation durations are determined in parafoveal viewing, called *forced fixations* (Schotter 2018). In cases where readers received a similar/plausible preview of the target, readers may make a short single fixation on the now dissimilar/implausible target, even when their attention has already shifted towards upcoming words, often producing delayed effects (Schotter 2018). Thus, it is not always straightforward directly mapping current fixations to lexical processing, since semantic effects can be distributed across multiple fixations. We return to these points later as they have important implications for the interpretation of long‐latency fixation‐related brain potentials.

### 4.2 When Do Readers Access Semantic Information?

#### 4.2.1 Contextually‐Graded Parafoveal N400

Several electrophysiology studies using both non‐natural and natural paradigms also show evidence that semantic information can be extracted parafoveally in English (e.g., Barber et al. 2011). Stites et al. (2017) and Payne et al. (2019) showed that semantic expectancy modulates parafoveal N400 amplitude, a well‐characterized index of semantic access from long‐term memory (Kutas and Federmeier 2011). In these flanker RSVP studies, this graded N400 at the parafovea was subsequently facilitated when readers made a direct fixation on the target word. Co‐registration studies provide a stronger test case for parafoveal semantic processing in natural reading, and two studies have replicated this parafoveal N400 effect for a plausibility manipulation (Antúnez et al. 2021; Li et al. 2024). The current study replicates this finding that N400 plausibility effects come online parafoveally, eliciting stronger effects at pre‐target fixation, and extends their findings by showing intermediate, graded effects of expectancy. Specifically, by including both semantically unexpected and anomalous targets in the same experiment, we believe we are the first to demonstrate a contextually graded parafoveal N400 effect in natural reading in English, with larger effect sizes, earlier onsets, and more sustained anomaly effects compared to expectancy effects. The mass univariate analyses help contextualize these effects, showing that the parafoveal expectancy and anomaly effects have qualitatively different spatiotemporal profiles, with the anomaly effect appearing to be more distributed (i.e., earlier in onset, stronger in magnitude, and more sustained) in space and time than the expectancy effect.^13^ This suggests that readers are not merely applying a simple mismatch heuristic, such as the difference between the expected and received orthography of the upcoming word to elicit early N400 effects, because if that were the case, we should see qualitatively similar responses to the anomalous and unexpected but plausible target words.

#### 4.2.2 Foveal Attenuation of the N400

We also show that when readers subsequently fixate on the target word, this graded N400 effect appears to be attenuated, replicating past work with the flanker RSVP paradigm (Stites et al. 2017; Payne et al. 2019; C. Li et al. 2023). Indeed, we see the same kind of rich semantically graded N400 effects that are observed in foveal vision with standard RSVP paradigms and these effects are stronger time‐locked to the preview than the target. We argue that this data pattern suggests that readers can begin retrieving relevant semantic information before foveation and that this provides trans‐saccadic processing benefits when the word is subsequently fixated.

##### 4.2.2.1 Is the N400 Compatible With the Time‐Course of Eye‐Movements?

Many have speculated that the N400 effect occurs too late in timing to align with eye movements (e.g., Sereno and Rayner 2003; Dambacher and Kliegl 2007; Burnsky et al. 2023). However, in the present study we see no appreciable difference in N400 latency in natural reading, even though the process arises while the target word is in parafoveal vision (just like in eye‐movement studies). Indeed, the influence of the parafoveal N400 effect on the target word FRP (appearing as a potentially earlier modulation of the P2) is apparent with visual inspection of the FRP waveforms by comparing representative electrodes (Figure 1), the unrestricted mass univariate analyses (Figure S6, Materials S1), and scalp topographies (Figure S5, Materials S1) across pre‐target and target regions. The N400 effect appears to onset earlier in target regions, particularly in the contrasts against expected items, but this in fact is driven by component overlap due to continued activation from the N400 triggered during initial parafoveal viewing of the target. A similar finding has recently been reported in the semantic processing of objects in scenes. Coco et al. (2020) examined scene semantic consistency effects (i.e., whether a target object was consistent in meaning with the scene) using fixation‐related brain potentials. A scene consistency N300/N400 effect was observed when time‐locking to the first fixation on the target object. However, they show that this effect was *already visible* when time‐locking to the fixation just prior to the first fixation on the object (i.e., during saccade planning toward the object when it appears in extrafoveal vision). This effect continues throughout the first fixation on the object, leading to an earlier looking onset for the scene consistency effect.

### 4.3 Strengths of Using a Natural Reading Paradigm

One way to account for component overlap in FRP studies (and indeed any ERP study with rapid succession of stimuli) is to use regression methods to deconvolve FRPs time‐locked to successive fixations (e.g., Ehinger and Dimigen 2019). However, to fully isolate partially overlapping parafoveal and foveal N400 effects, an orthogonal manipulation of parafoveal and foveal word identity is necessary, for example using a boundary‐change design, such as N. Li et al. (2024). Importantly however, they report analyses directly comparing standard averaging to deconvolved FRPs (see their Materials S1) and find no difference in the condition effects on FRPs, which they argue indicates that their N400 results (which we conceptually replicate for valid implausible previews) were not caused by differences in temporal overlap.

Since we did not use a display change design, readers always received valid parafoveal preview information. Everything in the sentence preceding and following the target was held constant across conditions (i.e., only the target word differed by condition, and this remained constant throughout the trial). Some work shows that altering parafoveal representations during reading may change the nature of word processing. For example, in the flanker ERP literature, Stites et al. (2017) discuss how changing parafoveal information may reduce readers' treatment of parafoveal words as reliable cues. This difference may explain why studies with reliable parafoveal cues (as in the current study; see also Payne and Federmeier 2017; Payne et al. 2019; Li et al. 2023) show evidence of foveal facilitation of the N400, whereas studies with unreliable (changing) parafoveal cues do not (Barber et al. 2011; Schotter et al. 2023). In our study, readers do not get conflicting preview and target information, and we view this as a major strength of using naturalistic reading designs to examine parafoveal and foveal FRPs.

#### 4.3.1 On the Locus of Effects in Natural Reading Co‐Registration Studies

There are a rapidly growing number of co‐registration studies of reading that (like ours) include single target‐word manipulations in the absence of gaze‐contingent paradigms to explicitly manipulate parafoveal word identity. In these natural reading studies, the canonical analysis approach would be to time‐lock FRPs to the location of the experimental manipulation—the first fixation on the target word. However, as we demonstrated here in natural reading (and has been shown in scene perception; Coco et al. 2020), initial sensitivity to semantic effects is clearly detectable (and may even be stronger) in extrafoveal vision. If we had followed the canonical approach and not time‐locked our FRPs to pre‐target word fixations, we may have falsely concluded that the N400 was overall quite weak (i.e., only sensitive to anomalies and not unexpected words), and perhaps had an earlier onset during natural reading. In actuality, we observed larger and more robust effects of both manipulations when time‐locking to the pre‐target word, revealing a robust graded parafoveal N400 effect. Thus, when studying natural reading, even if the focus of the study is not parafoveal processing per se, it is still critical to examine activity preceding the target word. Under normal reading conditions (i.e., without boundary changes), parafoveal processing of the target *is occurring* and impacting semantic processing, leading to modulation of the N400 before the target word is first fixated. In other words, if analyses of semantic influences only focus on target fixations, then one is likely to miss parafoveal influences at best, and completely mischaracterize true parafoveal effects as target word effects at worst.

### 4.4 Do Prediction Violations Incur Additional Processing?

In this study, we were interested in comparing theories of predictive processing emerging from the ERP and ET literatures during natural reading. For example, Frisson et al. (2017) argue against the existence of prediction violation consequences based on null effects of constraint on unexpected target words in the eye‐movement record (see also Luke and Christianson 2016), which has been framed as a lack of prediction *costs* in this literature (for a recent discussion see Staub 2025). Their results for the unexpected vs. expected contrast in a constraining context closely match our observed results: we see that readers spent more time fixating unexpected targets compared to expected in all duration‐based measures. Likely, there's a concurrent influence from both facilitation when receiving the expected input from the context and additional processing when receiving a lexical candidate that does not match anticipatory expectations, which we cannot dissociate in our current design. We also observed that prediction violations were facilitated relative to anomalous words in eye movements, suggesting much stronger disruptions when receiving a lexical candidate that is completely anomalous compared to one that is plausible but unexpected.

In contrast, a substantial literature links anterior positivities elicited by prediction violations to unpredicted (yet plausible) words to processing changes associated with high‐level and active comprehension processes, such as updating or revising semantic representations at multiple levels of analysis (e.g., Kuperberg et al. 2020; Federmeier 2022). Indeed, in the current study we observed a late anteriorly distributed positive component following the N400 that was larger for unexpected yet plausible target words compared to anomalous words time‐locked to first fixations on the target word. This effect is consistent with prior work using single‐word RSVP methods showing evidence for frontally distributed neural activity sensitive to prediction violations (e.g., DeLong et al. 2014; Federmeier et al. 2007; Kuperberg et al. 2020). At a minimum, this component distinguishes between plausible prediction violations and outright semantic anomalies that cannot be readily integrated into building a message‐level semantic representation. While there was a significant positive cluster in the direction of the frontal positivity time‐locking to the first fixation on the pre‐target word (unexpected minus anomalous contrast), the relatively short duration of this effect appears more consistent with continued distributed parafoveal N400 activation, rather than a genuine parafoveal anterior positivity.^14^ These findings suggest that the processes associated with the anterior positivity are likely not initiated based on parafoveal processing, and may require direct foveation, in contrast to the N400, consistent with similar prior claims on the LPC (Payne et al. 2019; Milligan et al. 2023; Schotter et al. 2023).

Collectively, the current findings indeed suggest there is additional processing associated with unexpected words that violate predictions in naturalistic reading, but that these processes may not be detrimental to overall comprehension fluency in real‐time eye movement planning and execution, at least at the stage of fixating pre‐target or target words. Critically, our current findings suggest that these differences in sensitivity to prediction violations across EEG and eye movements clearly are not due to differences in stimuli, participants, nor methodological differences, given our co‐registration design. Overall, these findings instead appear to suggest that ERP/FRP measures may be more sensitive to processing of new unexpected information than eye movements (at the target word). Future analyses directly yoking anterior positivity variation to reading behavior (e.g., regressions: Metzner et al. 2017; reading time: Payne and Federmeier 2017) may allow us to more directly probe the neural mechanisms of behavioral consequences to encountering unexpected input in future work.

### 4.5 How Do Readers Process Semantic Anomalies?

Finally, we show evidence for an LPC to anomalous targets when time‐locking to first fixations on the target word. The LPC has been linked to anomaly detection and/or integration attempts (e.g., revision or re‐analysis processes) in establishing coherent meaning representations (Brouwer et al. 2017; Kuperberg et al. 2020). At the target word, there was a significant difference between the anomalous minus unexpected contrast in the direction of a canonical LPC over posterior electrodes, and the constrained mass univariate analyses showed two significant positive clusters throughout most of our 600–1000 ms window over posterior electrodes (see Figure 3, bottom).

It is important to note that we did observe a small and short (less than 100 ms) significant cluster for the same A—U contrast when time‐locking to the pre‐target word. However, because this effect occurred so late in the epoch (over 900 ms after first fixations on the pre‐target word), and thus after the eyes have, on average, moved onto (and indeed past) the target word at this point, it is likely that this effect reflects the subsequent foveal LPC that is clearly observed in the target analyses. These findings are thus more compatible with a foveal origin to the LPC (Payne et al. 2019; Li et al. 2023; Schotter et al. 2023). Although it does not reflect a canonical LPC effect in parafoveal vision, there was also a significant contrast between anomalous and expected conditions time‐locked to the pre‐target word in posterior electrodes, such that the *expected* condition was more positive than anomalous. Schotter et al. (2023) using flanker RSVP observed this same reversed pattern in the LPC region of interest at parafoveal target viewing. Given the direction of this effect, the clear pattern of a more sustained parafoveal N400 (see Figure 1), and variability in fixation durations, it is likely that the effect we report here is a continuation of the N400 effect. At the same time, previous work experimentally manipulating presentation rate (Wlotko and Federmeier 2015), as well as work allowing for self‐pacing of presentation rate (Payne and Federmeier 2017) has shown little evidence that variability in reading time has an impact on N400 onset latency or duration. Another interpretation is that it could be related to previously observed centro‐parietal sustained negativities (Ditman et al. 2007; Hagoort 2003; Osterhout and Holcomb 1992). Why parafoveal N400 effects appear more sustained in natural reading remains a question for future work.

### 4.6 Role of Online Plausibility Judgment Task

Prior work suggests LPC amplitude and latency are highly sensitive to task demands and response time (e.g., Payne et al. 2019; Sassenhagen et al. 2014). Payne et al. (2019) using flanker RSVP showed that a foveal LPC was elicited from incongruent targets, but only when readers made an explicit plausibility judgment. In contrast, the parafoveal and foveal N400 effects were invariant to task effects. Although we mirrored this design in the present study to explicitly enhance the LPC, the magnitude of this effect was smaller in our study than in prior studies using both fixed‐gaze flanker paradigms (Payne et al. 2019; Schotter et al. 2023) and at least one prior study using co‐registration (Metzner et al. 2017).

Additionally, the plausibility judgment task may differ from the canonical eye‐tracking task of reading to answer comprehension questions (as has been reported in several prior eye‐tracking studies where task demands were explicitly manipulated, see for example, White et al. 2015; Kaakinen and Hyönä 2010; Schotter et al. 2014; Duggan and Payne 2009; Strukelj and Niehorster 2018). Nevertheless, for our eye‐tracking measures, we did not observe evidence that there were major task‐driven differences, since all of our reported eye‐tracking effects appear to replicate what has been found in prior eye‐tracking studies on predictability and plausibility effects using a comprehension question task. Similarly, we do not believe that the anterior positivity was influenced by our online task, given that Lai et al. (2023) did not find differences in the anterior positivity to prediction violations when asked to actively predict compared to passive comprehension (see also Payne et al. 2019).

### 4.7 Variability in Long‐Latency Potentials

More broadly, emerging work shows that some late long‐latency potentials are generally more variable than the N400, even in traditional RSVP studies. For example, Kim et al. (2024) show via resampling simulations that there are differences in the power needed to detect N400 compared to P600 effects (to syntactic violations) due to this variability. This issue may become exacerbated with the additional measurement error and SNR of FRPs in natural reading compared to fixed‐gaze RSVP paradigms (Payne et al. 2019; Metzner et al. 2017), which afford greater experimental control at the cost of reduced ecological validity. While co‐registration work is in its infancy, newer tools for quantifying data quality (e.g., ERP measurement error; Luck et al. 2021) and emerging simulation‐based approaches to quantifying power in co‐registration paradigms (Schepers et al. 2025) will be critical for the effective design of future co‐registration studies.

## 5 Conclusions and Future Directions

In summary, our eye‐movement and electrophysiological results suggest that readers show multiple effects of sensitivity to predictability and plausibility that unfold differentially across the visual field, in the absence of artificial manipulations. We see clearly that, while the N400 is strongest parafoveally, the anterior positivity and LPC are only reliably observed at foveal target fixation, replicating and extending prior work in non‐naturalistic flanker reading studies (e.g., Payne et al. 2019; Schotter et al. 2023). The present findings support the idea that some semantic processes can be initiated parafoveally—such as early semantic retrieval processes associated with the N400—whereas other high‐level processes, linked to post‐lexical processes associated with semantic updating, reanalysis and integrative processing as indexed by the anterior positivity and LPC, seem to be more strongly yoked to foveal processing and may require direct fixation to engage. In combination with studies of natural reading, future work in predictive coding modeling may be a fruitful avenue towards better adjudicating between conflicting theoretical models across literatures (and as a way to formalize verbal theories as in Nour Eddine et al. 2024). Our findings replicated and extended several existing findings across both the eye‐tracking and electrophysiology literatures, including the first demonstration of contextually graded parafoveal N400 effects and foveal anterior positivity prediction violation effects in natural reading. Readers are sensitive very early on to both predictability and plausibility expectation violations but these effects persist for hundreds of milliseconds post‐target word processing, well after the eyes have moved on to other words. These findings therefore have important implications for studies exclusively focusing on eye movements to establish the existence, temporal locus, or duration of effects in natural reading.

## Author Contributions

**Allyson Copeland:** conceptualization, data curation, formal analysis, methodology, software, visualization, writing – original draft, writing – review and editing. **Brennan R. Payne:** conceptualization, methodology, project administration, resources, supervision, writing – review and editing.

## Conflicts of Interest

The authors declare no conflicts of interest.

## Supporting information


**Data S1:** psyp70173‐sup‐0001‐DataS1.zip.

## Data Availability

The data that support the findings of this study are available from the corresponding author upon reasonable request.

## Endnotes

^1^
See Materials S1 for more participant information.

^2^
We ran an updated sensitivity analyses with 43 subjects, and with 162 items, we are powered at 0.80 to detect an effect size of 0.335.

^3^
The preregistration stated that the LPC would use the same spatial ROI as the N400, but the mean amplitude analyses conducted used a slightly more posterior set of electrodes, given the more posterior distribution of the LPC often observed in the literature (Van Petten and Luka 2012).

^4^
Bayes factor analyses were considered in the preregistration as they help in interpreting evidence in favor of a null, but since we did not have any major findings that rested on interpreting null results, we did not include these as supplementary analyses in order to streamline reporting.

^5^
We originally conducted mass univariate analyses on all condition contrasts for the entire 0–1200 ms epoch. Based on thoughtful reviewer comments, we have moved these original exploratory analyses to Materials S1 and added the constrained mass univariate analyses described here.

^6^
As a partial deviation from the preregistration to streamline results reporting in response to reviewer feedback, we report most null pre‐target results, as well as single fixation duration and total reading time measures in Materials S1 only.

^7^
Tables with summarized parameter estimates and summarized amplitude means and standard errors for the three reported fixation‐related components across conditions and word regions can be found in Materials S1.

^8^
Raster plots for all significant clusters are included in Materials S1.

^9^
To better visualize this parafoveal activation, see scalp topography contrasts in Figure S5 and the raster plot for this contrast in Materials S1.

^10^
To better visualize this effect, see Figure S5 for the scalp topographies and the raster plot for this contrast in Materials S1.

^11^
Summarized parameter estimates for each a priori eye‐tracking measure and region of interest (including those that are reported here) can be found in Materials S1.

^12^
For duration‐based measures, the log‐transformed outcome models showed the same pattern of results but given that the results were unchanged and that the effect sizes are more easily interpreted in original raw (ms) units, the log‐transformed models are not reported here for brevity.

^13^
For better visualization of this graded effect, see additional mass univariate raster plots in Materials S1, particularly Figure S6A–C. Note that this is not a clear A > U > E pattern because there were no significant A versus U contrasts in either the a priori or constrained mass univariate analyses.

^14^
The unrestricted mass univariate analyses illustrate this clustering of the frontal electrodes with the centro‐parietal electrodes (see Figure S6C).
