# Supplementary material for: Co‐Registered Eye‐Movements and Brain Potentials Reveal Multiple Effects of Context Across the Visual Field in Natural Reading
Source: Psychophysiology. 2025 Nov 17;62(11):e70173. doi: 10.1111/psyp.70173 (PMC12623278; doi:10.1111/psyp.70173)
Supplement: Supplementary file 1 — Data S1: psyp70173‐sup‐0001‐DataS1.zip. [file PSYP-62-e70173-s001.zip › psyp70173-sup-0001-Supinfo01.pdf]

**Supplementary Eye-Tracking Materials**

Based on reviewer suggestions, in order to streamline the results reporting of our many preregistered eye-tracking measures, we decided to move some less critical measures to supplementary materials. All results that were moved from the article have been included below.

**Additional Eye-Tracking Results**

The results below have been divided into pre-target and target regions. Many pre-target measures were non-significant and thus did not need to be included. The target measures that were moved were relatively redundant with measures that are reported in the article.

<u>Pre-target Region</u>

We report below the results for first-pass probability of skipping, first-pass probability of regressing, first-pass probability of re-fixation, single fixation duration, regression path duration, and total reading time at the pre-target word.

At the pre-target word, there were no significant main effects of condition for single fixation duration ($\chi^2$ (2) = 0.69, $p$ = 0.71). There was a significant main effect of condition for regression path duration ($\chi^2$ (2) = 6.24, $p$ = 0.04) but pairwise contrasts were not significant. For total reading time at the pre-target word, there was a significant main effect of condition ($\chi^2$ (2) = 100.18, $p < 0.001$) and pairwise contrasts showed that pre-target words that preceded anomalous targets (*EMM* = 503 ms) were fixated for 141 ms longer than in the expected condition (*EMM* = 362; $t$ (5515) = 10.01, $p < 0.0001$) and 80 ms longer than in the unexpected condition (*EMM* = 423 ms; $t$ (5512) = 5.78, $p < 0.0001$). Unexpected targets were fixated for 60 ms longer than expected targets ($t$ (5511) = -4.28, $p$ = 0.0001). Note that because total reading time is not a first-pass measure, these effects are not interpreted as reflecting parafoveal processing of the target.

At the pre-target word, there was no significant main effect of condition for first-pass probability of skipping ($\chi^2$ (2) = 0.07, $p$ = 0.97) first-pass probability of regressing ($\chi^2$ (2) = 0.74, $p$ = 0.69), or first-pass probability of re-fixation at the pre-target word ($\chi^2$ (2) = 4.59, $p$ = 0.10).

Target Region

We report below the results for single fixation duration and total reading time at the target.

At the target word, single fixation duration there was a significant main effect of condition ($\chi$2 (2) = 132.87 $p$ < 0.001). Pairwise contrasts showed that anomalous targets (*EMM* = 281 ms) were fixated for 54 ms longer than expected targets (*EMM* = 228; $t$ (1964) = 11.71, $p$ < 0.0001) and 37 ms longer than unexpected targets (*EMM* = 245 ms; $t$ (1979) = 7.79, $p$ < 0.0001). Unexpected targets were fixated for 17 ms longer than expected targets $t$ (1949) = -4.28, $p$ = 0.0001).

For total reading time at the target word, there was a significant main effect of condition ($\chi^2$ (2) = 428.08, $p$ < 0.001). Pairwise contrasts showed that anomalous targets (*EMM* = 575 ms) were fixated for 263 ms longer than expected targets (*EMM* = 312; $t$ (5385) = 20.14, $p$ < 0.0001) and 193 ms longer than unexpected targets (*EMM* = 382 ms; $t$ (5380) = 15.16 $p$ < 0.0001). Unexpected targets were fixated for 70 ms longer than expected targets ($t$ (5384) = -5.27, $p$ < 0.0001).

**Eye-Tracking Summary Tables**

The summary tables below include all preregistered eye-tracking measures. Table 2a is an expanded version of Table 2 in the article, which additionally includes single fixation duration and total reading time. Table 3 includes parameter estimates for all eye-tracking outcomes.

**Table 2a.** Means (and standard errors) for all reading measures by condition for pre-target and target words.

| | Pre-Target | | | Target | | |
|---|---|---|---|---|---|---|
| | Anomalous | Unexpected | Expected | Anomalous | Unexpected | Expected |
| Single Fixation Duration | 235 (3) | 235 (3) | 233 (3) | 278 (6) | 242 (3) | 226 (3) |
| First Fixation Duration | 237 (2) | 237 (2) | 234 (2) | 271 (3) | 242 (3) | 228 (3) |
| Gaze Duration | 262 (3) | 257 (3) | 257 (3) | 300 (4) | 257 (3) | 239 (3) |
| Regression Path Duration | 319 (6) | 302 (5) | 307 (6) | 403 (10) | 321 (8) | 289 (8) |
| Total Reading Time | 512 (16) | 433 (10) | 376 (9) | 583 (14) | 391 (9) | 322 (10) |
| Probability of Skip | 0.33 (0.01) | 0.33 (0.01) | 0.33 (0.01) | 0.45 (0.01) | 0.46 (0.01) | 0.49 (0.01) |
| Probability of Regressing | 0.11 (0.01) | 0.10 (0.01) | 0.10 (0.01) | 0.12 (0.01) | 0.09 (0.01) | 0.07 (0.01) |
| Probability of Re-Fixating | 0.11 (0.01) | 0.09 (0.01) | 0.10 (0.01) | 0.10 (0.01) | 0.06 (0.01) | 0.05 (0.01) |

*Note:* Values are reported in milliseconds for duration-based measures and probabilities for probability-based measures.

**Table 3.** Parameter estimates for each eye-tracking measure separately for pre-target and target words.

| | Pre-Target | | | Target | | |
|---|---|---|---|---|---|---|
| | Est. (β) | SE | *t*-/z-value | Est. (β) | SE | *t*-/z-value |
| Single Fixation Duration | | | | | | |
| Anomalous - Expected | 2.32 | 3.57 | 0.65 | 53.8 | 4.59 | 11.71*** |
| Anomalous - Unexpected | -0.14 | 3.66 | -0.04 | 36.7 | 4.71 | 7.79*** |
| Expected - Unexpected | -2.46 | 3.28 | -0.75 | -17.1 | 4.00 | -4.28** |
| First Fixation Duration | | | | | | |
| Anomalous - Expected | 2.90 | 2.54 | 1.14 | 42.9 | 3.42 | 12.56*** |
| Anomalous - Unexpected | -0.56 | 2.54 | -0.22 | 28.7 | 3.37 | 8.54*** |
| Expected - Unexpected | -3.46 | 2.54 | -1.36 | -14.2 | 3.43 | -4.13** |
| Gaze Duration | | | | | | |
| Anomalous - Expected | 6.09 | 3.59 | 1.70 | 60.6 | 4.32 | 14.04*** |
| Anomalous - Unexpected | 4.42 | 3.58 | 1.23 | 43.2 | 4.25 | 10.15*** |
| Expected - Unexpected | -1.67 | 3.59 | -0.47 | -17.5 | 4.33 | -4.03** |
| Regression Path Duration | | | | | | |
| Anomalous - Expected | 13.80 | 7.08 | 1.95 | 116.0 | 10.08 | 11.51*** |
| Anomalous - Unexpected | 16.81 | 7.19 | 2.34^ | 82.6 | 9.93 | 8.32*** |
| Expected - Unexpected | 3.01 | 7.50 | 0.40 | -33.4 | 10.12 | -3.30** |
| Total Reading Time | | | | | | |
| Anomalous - Expected | 140.6 | 14.1 | 10.01*** | 262.6 | 13.0 | 20.14*** |
| Anomalous - Unexpected | 80.2 | 13.9 | 5.78*** | 193.1 | 12.7 | 15.16*** |
| Expected - Unexpected | -60.4 | 14.1 | -4.28*** | -69.6 | 13.2 | -5.27*** |
| Probability of Skip | | | | | | |
| Anomalous - Expected | -0.02 | 0.07 | -0.24 | -0.17 | 0.06 | -2.70* |
| Anomalous - Unexpected | 0.00 | 0.07 | 0.03 | -0.04 | 0.06 | -0.70 |
| Expected - Unexpected | 0.01 | 0.07 | 0.21 | 0.12 | 0.06 | 2.00 |
| Probability of Regressing Out of Region | | | | | | |
| Anomalous - Expected | 0.05 | 0.11 | 0.50 | 0.63 | 0.13 | 4.93*** |
| Anomalous - Unexpected | 0.10 | 0.11 | 0.88 | 0.41 | 0.12 | 3.48** |
| Expected - Unexpected | 0.04 | 0.11 | 0.37 | -0.22 | 0.14 | -1.57 |
| Probability of Re-Fixating | | | | | | |

| | | | | | | |
|---|---|---|---|---|---|---|
| Anomalous - Expected | 0.12 | 0.11 | 1.10 | 0.85 | 0.13 | 6.36*** |
| Anomalous - Unexpected | 0.24 | 0.11 | 2.21^ | 0.58 | 0.12 | 4.82*** |
| Expected - Unexpected | 0.12 | 0.11 | 1.09 | -0.26 | 0.15 | -1.81 |

*Note*: ^$p$<0.1, * $p$<.05, ** $p$<.01, *** $p$<.001; Probability-based measures are reported on the log odds ratio scale.
