# Supplementary material for: Co‐Registered Eye‐Movements and Brain Potentials Reveal Multiple Effects of Context Across the Visual Field in Natural Reading
Source: Psychophysiology. 2025 Nov 17;62(11):e70173. doi: 10.1111/psyp.70173 (PMC12623278; doi:10.1111/psyp.70173)

Supplementary Fixation-Related Potential Materials

Based on reviewer suggestions, we updated our mass univariate analyses with the constrained mass univariate analyses (as in Fields & Kuperberg, 2020) that are now in the article. We decided to change the mass univariate analyses to an ROI- restricted mass univariate analysis to 1) streamline results reporting and 2) increase sensitivity for the late positivities. Our original mass univariate approach (which was conducted across all time-points and all channels) likely resulted in an overly conservative test (a known issue in mass univariate approaches), making the LPC undetectable initially. We have included below our original mass univariate analyses and corresponding figures.

Original Mass Univariate Analyses

Exploratory Mass Univariate FRP Analyses. To detect reliable differences between neural processing of the expected, unexpected, and anomalous words outside of our target ROIs, FRPs from the three pairwise contrasts of these conditions described above were additionally submitted to a cluster-based permutation test (Maris & Oostenveld, 2007) based on the cluster mass statistic (Bullmore et al., 1999), using a family-wise alpha of 0.05. All time points between 0 and 1200 ms (601 time points) at all 31 scalp electrodes (online reference excluded) were included in the test (i.e., 18631 total comparisons) and any electrodes within approximately 5.44 cm of one another were considered spatial neighbors. Repeated measure t-tests were performed for each comparison using the using the original data and 2500 random within-participant permutations of the data. For each permutation, all t-scores corresponding to uncorrected p-values of 0.05 or less were formed into clusters. The sum of t-scores in each cluster is the "mass" of that cluster and the most extreme cluster mass in each of the 2501 sets of tests was recorded

and used to estimate the distribution of the null hypothesis. These analyses were conducted via the Mass Univariate ERP Toolbox (Groppe, Urbach, & Kutas, 2011).

Exploratory Mass Univariate FRP Results.

To evaluate more extensive spatiotemporal dynamics than we hypothesized a priori, we conducted exploratory mass univariate analyses. Figure 5 (see below) shows scalp topographies for each pairwise contrast across pre-target and target interest areas in fixed time windows from 300 – 1200 ms post fixation onset (replicating DeLong et al., 2014, Fig 4). This figure helps to visualize the time-course and scalp distribution for each FRP effect of interest. Some of the expected effects, including the LPC, appear to have maxima that are outside of our a priori regions of interest. Visual inspection of this figure suggests that the LPC may appear later in time than the a priori selected ROI, as a more positive posterior distribution is present between 800-1000ms. Thus, we conducted exploratory mass univariate analyses (Groppe et al., 2011) to examine whether such apparent effects are statistically robust. We conducted cluster-based permutation tests on each condition contrast in pre-target and target regions.

Figure 6 (see below) shows raster plots of significant clusters for each cluster-based permutation test. Time-locked to the pre-target word, permutation tests indicated that there was a single significant cluster in each contrast (unexpected - expected, anomalous - expected, and anomalous - unexpected). The negative direction and time-window of these clusters are consistent with the parafoveal N400 effect we analyzed in centro-parietal regions, and there was also some significant activation in frontal electrodes. The observed cluster for the unexpected – expected contrast is consistent with an N400 expectancy effect onsetting in parafoveal vision. This cluster shows activation primarily in central and posterior electrodes extending from approximately 320 to 678 ms. The observed cluster for the anomalous – unexpected contrast is

consistent with the parafoveal N400 anomaly effect, with activation primarily in central and posterior electrodes extending from approximately 436 to 868, with a shift towards frontal electrodes towards the end of the time window. It is clear that, despite the null contrast between unexpected and anomalous targets in our ROI-based N400 analysis reported above, the mass univariate analyses clearly reveal significant differences in the parafoveal N400 such that the anomalous condition is more negative than the unexpected throughout a broad spatio-temporal cluster (see Figure 6B). The observed cluster for the anomalous – expected contrast is consistent with this contextually graded parafoveal N400, with activation in central and posterior electrodes, and continued activation in frontal electrodes.

Time-locked to the target word, permutation tests indicated that there was a single significant cluster in U-E and A-E condition contrasts, and 2 significant negative clusters in the A-U contrast. Consistent with a target N400, the observed cluster for unexpected - expected extended from approximately 82 to 360 ms primarily in centro-posterior electrodes. The observed cluster for anomalous - expected targets in centro-posterior electrodes is also consistent with the N400, and there was also activation in frontal electrodes. Consistent with the anterior positivity, the observed clusters for anomalous - unexpected targets extended from approximately 302 to 1200 ms (with a small break at 832 to 862 ms) with most activation in frontal channels, but also some in central channels approximately 310 to 400 ms. Overall, with these mass univariate analyses we see evidence for an N400 time-locked to both pre-target and target words, and an anterior positivity time-locked to target words (shown in the A-U raster plot as a negative difference), but no evidence of a statistically significant canonical LPC time locked to the pre-target or target word, counter to our predictions and our a priori ROI-based analyses.

Supplementary FRP Tables & Figures

The tables below are summarized information about the a priori mean amplitude analyses we conducted for the three fixation-related potential components. Figure 5 shows scalp topographies for all condition contrasts, meant to be comparable to DeLong et al. (2014) Figure 4. Figure 6 shows the original exploratory mass univariate raster plots for each condition contrast.

Table 4. Means (and standard errors) for fixation-related potential amplitude by condition and region of interest.

	Pre-Target			Target		
	Anomalous	Unexpected	Expected	Anomalous	Unexpected	Expected
N400	-1.55 (0.25)	-1.18 (0.21)	-0.23 (0.21)	0.36 (0.21)	0.55 (0.20)	1.11 (0.22)
Anterior Positivity	0.25 (0.19)	0.90 (0.21)	0.95 (0.22)	0.15 (0.22)	1.24 (0.21)	0.80 (0.19)
Late posterior complex	0.64 (0.23)	0.94 (0.17)	1.53 (0.18)	2.43 (0.17)	1.85 (0.19)	2.41 (0.17)

Note: Values are reported in µV.

Table 5. Parameter estimates of contrasts between conditions for each fixation-related potential at the pre-target and target word.

	Pre-Target			Target		
	Est. (β)	SE	*t*-value	Est. (β)	SE	*t*-value
N400						
Anomalous - Expected	-1.32	0.32	-4.10***	-0.75	0.30	-2.54*
Anomalous - Unexpected	-0.37	0.32	-1.15	-0.20	0.30	-0.66
Expected - Unexpected	0.95	0.32	2.95*	0.55	0.30	1.88
Anterior Positivity						
Anomalous - Expected	-0.70	0.30	-2.36^	-0.65	0.30	-2.17^
Anomalous - Unexpected	-0.65	0.30	-2.19^	-1.08	0.30	-3.62**
Expected - Unexpected	-0.05	0.30	-0.17	-0.43	0.30	-1.45
Late posterior complex						
Anomalous - Expected	-0.89	0.27	-3.33**	-0.02	0.24	0.08
Anomalous - Unexpected	-0.19	0.27	-1.09	0.58	0.24	2.44*
Expected - Unexpected	0.60	0.27	2.24^	0.56	0.24	2.37^

Note: ^ p<.10, * p<.05, ** p<.01, *** p<.001.

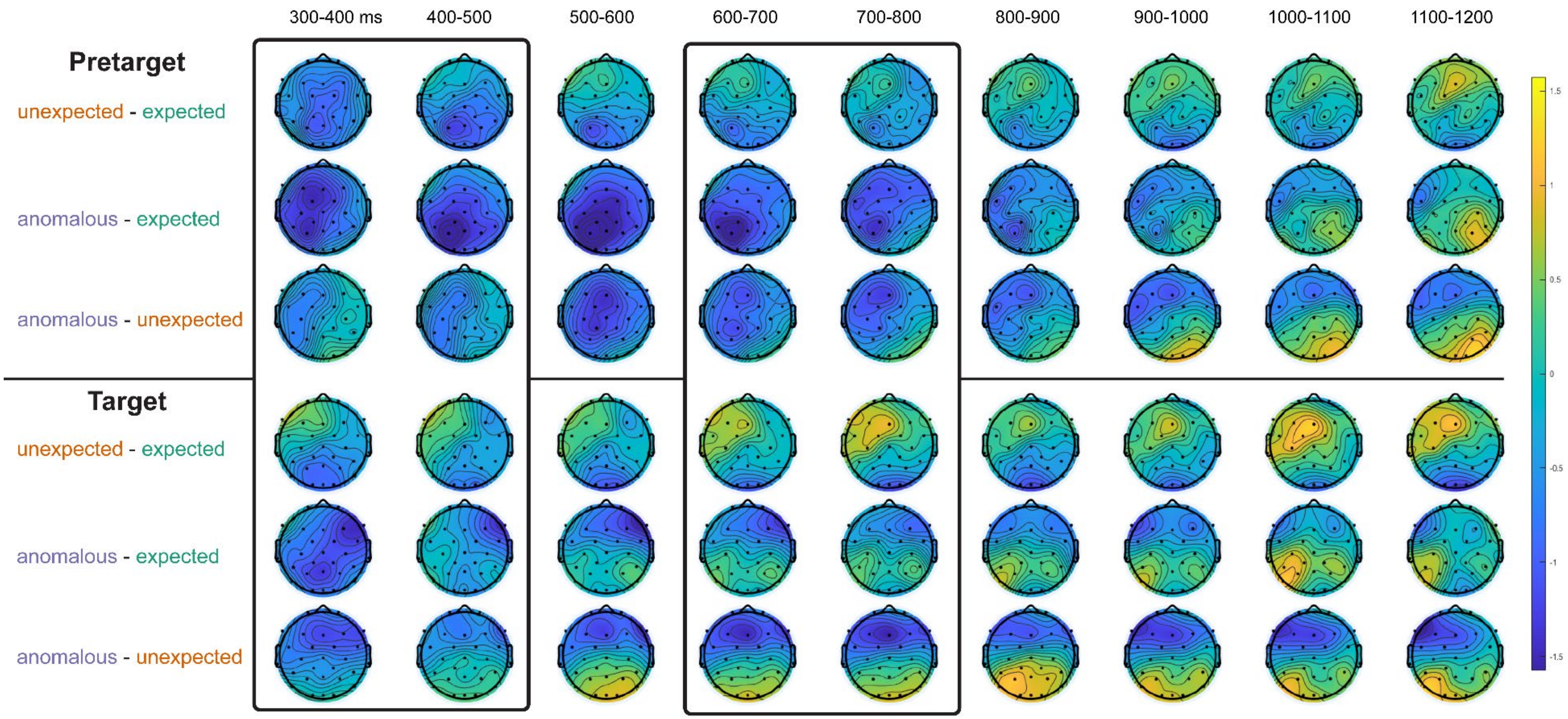

Figure 5. Scalp topography maps from 300 to 1200 ms for each condition contrast across pre-target and target regions. A priori interest areas are boxed.

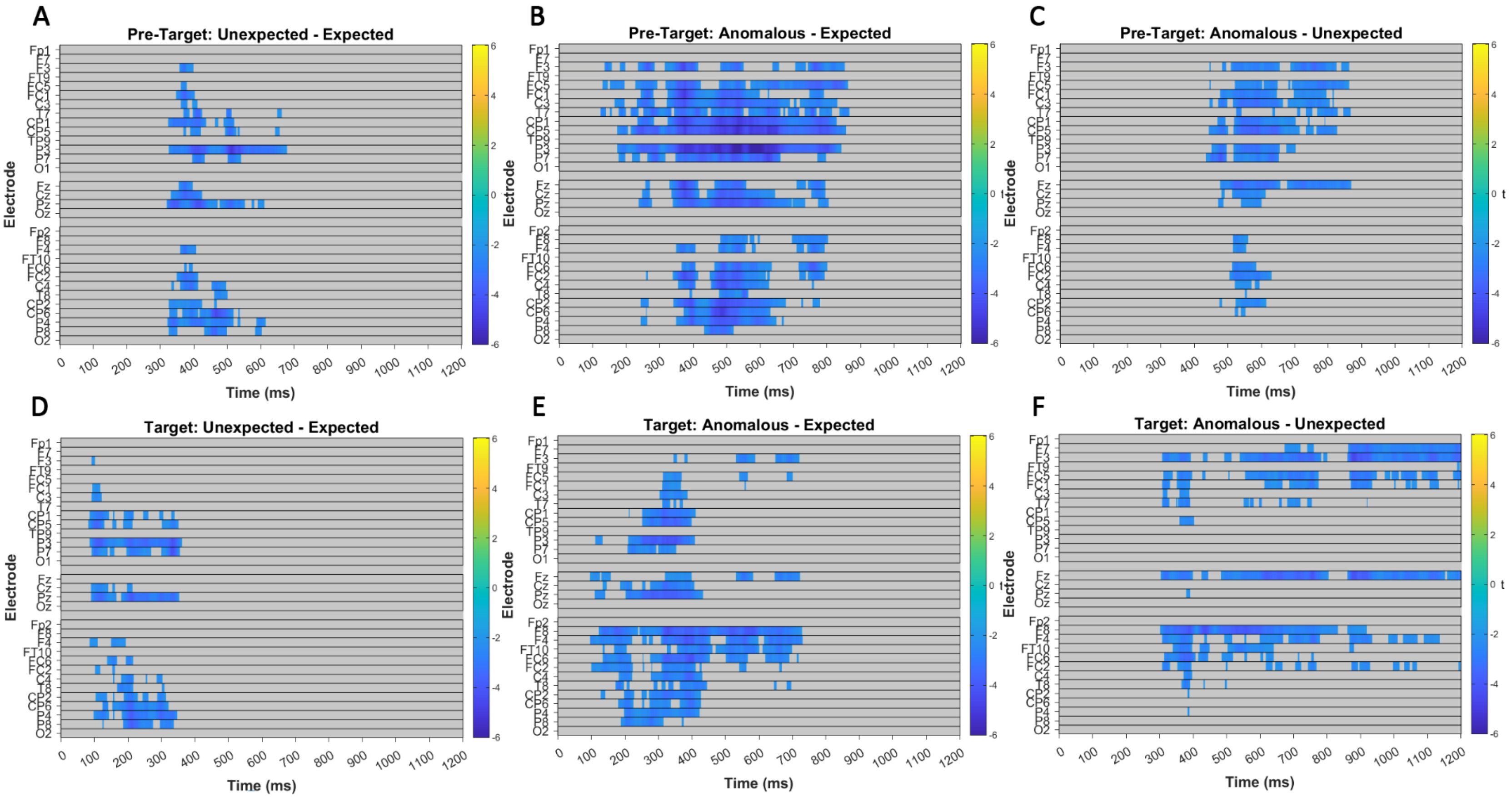

Figure 6. Mass univariate raster plots across word regions for each condition contrast. Only significant clusters are plotted.

Constrained Mass Univariate Raster Plots

Included below are the raster plots for all significant clusters that were reported in the manuscript. The target frontal positivity and LPC A vs U constrasts are additionally included in Figure 3.

<u>N400 spatiotemporal ROI</u>

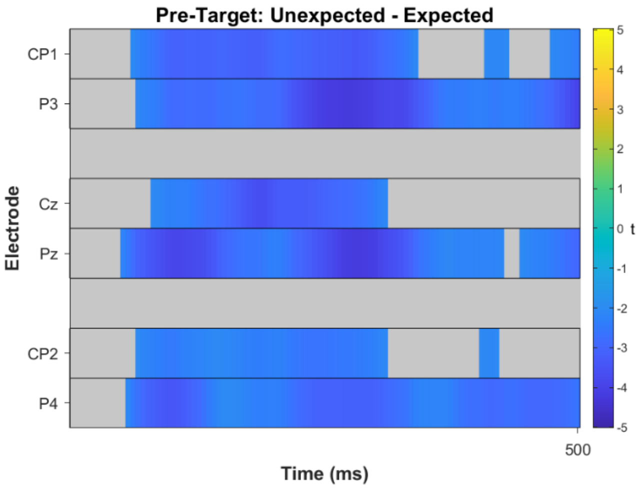

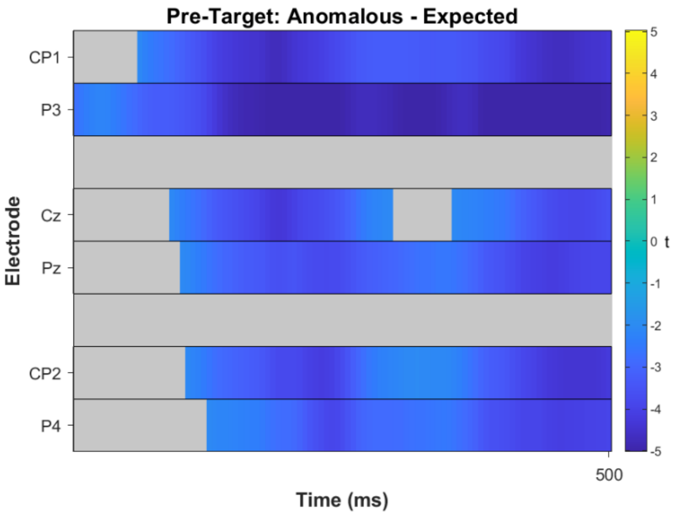

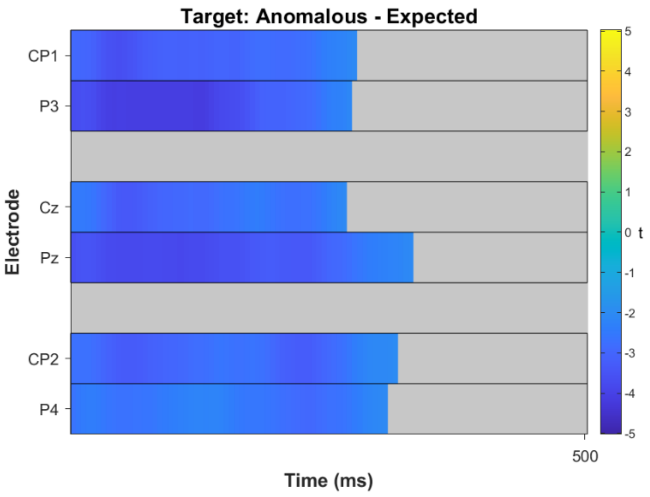

Anterior Positivity spatiotemporal ROI

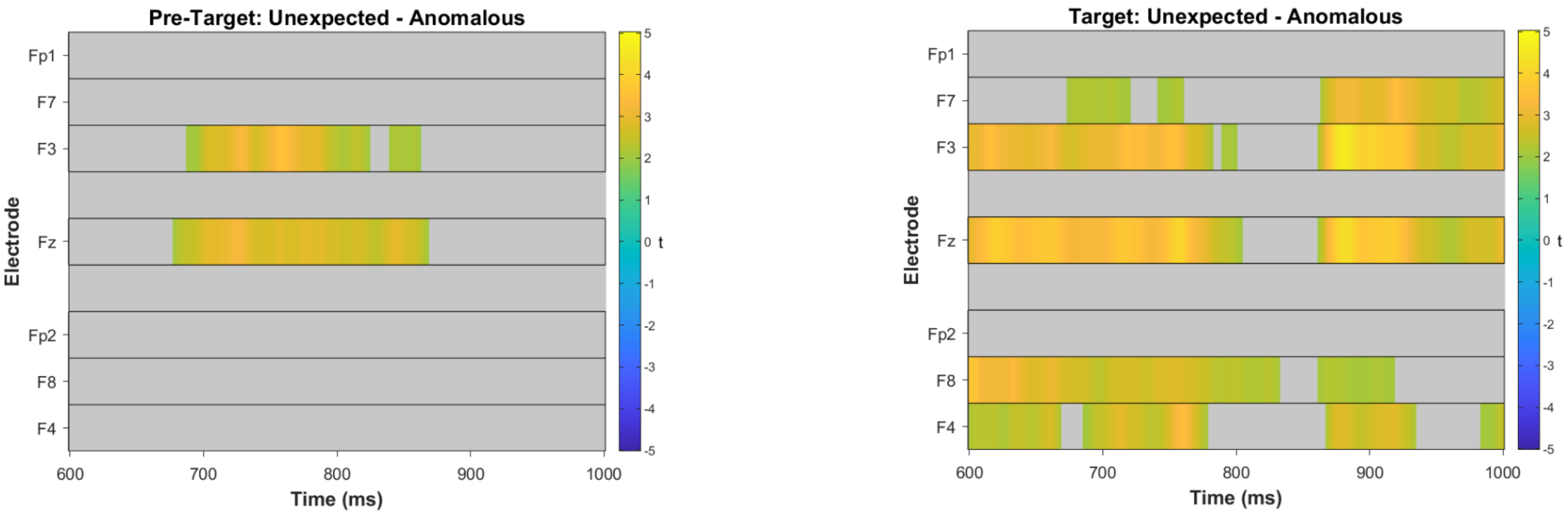

LPC spatiotemporal ROI

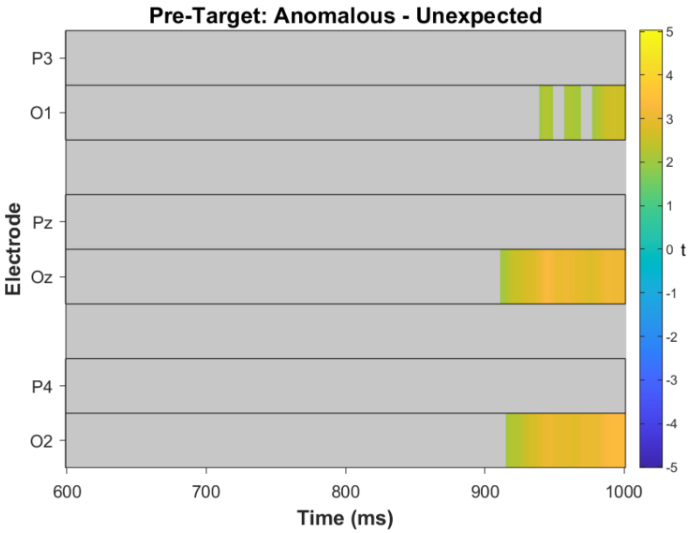

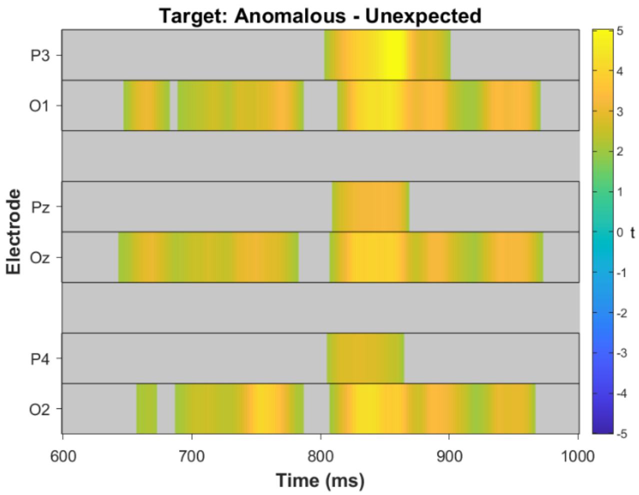

Supplement: Supplementary file 1 — Data S1: psyp70173‐sup‐0001‐DataS1.zip. [file PSYP-62-e70173-s001.zip › psyp70173-sup-0002-Supinfo02.pdf]
