# Supplementary material for: Co‐Registered Eye‐Movements and Brain Potentials Reveal Multiple Effects of Context Across the Visual Field in Natural Reading
Source: Psychophysiology. 2025 Nov 17;62(11):e70173. doi: 10.1111/psyp.70173 (PMC12623278; doi:10.1111/psyp.70173)
Supplement: Supplementary file 1 — Data S1: psyp70173‐sup‐0001‐DataS1.zip. [file PSYP-62-e70173-s001.zip › psyp70173-sup-0003-Supinfo03.pdf]

Supplementary Methods Information

Electrophysiology Processing.

Implementation Details for OPTICAT.

First, a stricter bandpass filter (3-100 Hz) was applied to EEG channels only for ICA weight calculation (not eventual FRP analyses). Following OPTICAT, a training dataset was created for each subject that is overweighted for eye movement events by appending 30% additional EEG epochs surrounding saccades (20 ms prior to 10 ms after), determined independently via the eye tracking data. The extended infomax ICA algorithm from EEGLAB was then run on the EEG channels in the overweighted training data. ICA weights were then transferred to the original EEG files. Ocular components were then identified by first taking a ratio of the variance of each IC's activation during saccades to activation during fixations and marking those whose ratio exceeded 1.1 (see Dimigen, 2020; Plöchl et al., 2012 for more detail). To additionally confirm ocular components, the *ICLabel* classifier, a classifier trained on expertly labeled ICA data that automatically labels independent components corresponding to their likely sources (e.g., brain, eye, muscle, line noise), was applied to each dataset (Pion-Tonachini et al., 2019). Recommendations from both information sources, along with visual inspection of the scalp topography, time-series, and power spectrum plots were conducted for each subject to determine which ocular components to remove. Once ocular ICs were identified and removed (M = 1.7 ICs removed per subject, range = 1-2), the dataset was saved for subsequent processing.
