# Supplementary material for: Co‐Registered Eye‐Movements and Brain Potentials Reveal Multiple Effects of Context Across the Visual Field in Natural Reading
Source: Psychophysiology. 2025 Nov 17;62(11):e70173. doi: 10.1111/psyp.70173 (PMC12623278; doi:10.1111/psyp.70173)
Supplement: Supplementary file 1 — Data S1: psyp70173‐sup‐0001‐DataS1.zip. [file PSYP-62-e70173-s001.zip › psyp70173-sup-0004-Supinfo04.pdf]

Supplementary Participant Information

Detailed Participant Demographics.

50 young adults (18 – 32, *M* = 21.24 years old) from the University of Utah and Salt Lake City community participated in this study for either course credit or payment of $10 per hour. There were 34 female subjects and 16 males. 45 participants were White/Caucasian, 2 were Asian, 1 was Black, and 2 were Hispanic/Latino. 16 reported high school diploma or equivalent, and the remainder had some college or higher. All reported normal or corrected-to-normal vision (NA = 1). 1 subject reported mild red/green colorblindness. 1 subject reported loss of central vision in the left eye (all eye-tracking was recorded from the right eye). All subjects reported English as their native language (NA = 1), and 8 reported early language exposure to another language. 11 participants self-reported some history of mental health conditions including attention-deficit/hyperactivity disorder, bipolar II, social anxiety, dermatillomania, sensory processing disorder, depression, general anxiety, obsessive-compulsive disorder, seasonal affective disorder, and post-traumatic stress disorder. 4 subjects reported having a prior head injury with loss of consciousness. 1 subject self-reported being dyslexic and was excluded from analyses. 2 subjects were excluded due to technical errors during recording, and 4 subjects were excluded due to high missing data (> 60% in any condition). All subsequently reported analyses are from the remaining 43 subjects.
